# Design, synthesis and biological evaluation of novel benzodioxole derivatives as COX inhibitors and cytotoxic agents

**DOI:** 10.1186/s13065-020-00706-1

**Published:** 2020-09-07

**Authors:** Mohammed Hawash, Nidal Jaradat, Saba Hameedi, Ahmed Mousa

**Affiliations:** 1 Department of Pharmacy, Faculty of Medicine and Health Sciences, An-Najah National University, P.O. Box 7, Nablus, 00970 Palestine; 2 Department of Biomedical Sciences, Faculty of Medicine and Health Sciences, An-Najah National University, Nablus, 00970 Palestine

**Keywords:** Benzodioxole, COX, Ketoprofen

## Abstract

Non-steroidal anti-inflammatory drugs are among the most used drugs. They are competitive inhibitors of cyclooxygenase (COX). Twelve novel compounds (aryl acetate and aryl acetic acid groups) were synthesized in this work in order to identify which one was the most potent and which group was most selective towards COX1 and COX2 by using an in vitro COX inhibition assay kit. The cytotoxicity was evaluated for these compounds utilizing MTS assay against cervical carcinoma cells line (HeLa). The synthesized compounds were identified using FTIR, HRMS, ^1^H-NMR, and ^13^C-NMR techniques. The results showed that the most potent compound against the COX1 enzyme was 4f with IC_50_ = 0.725 µM. The compound 3b showed potent activity against both COX1 and COX2 with IC_50_ = 1.12 and 1.3 µM, respectively, and its selectivity ratio (0.862) was found to be better than Ketoprofen (0.196). In contrast, compound 4d was the most selective with a COX1/COX2 ratio value of 1.809 in comparison with the Ketoprofen ratio. All compounds showed cytotoxic activity against the HeLa Cervical cancer cell line at a higher concentration ranges (0.219–1.94 mM), and the most cytotoxic compound was 3e with a CC_50_ value of 219 µM. This was tenfold more than its IC_50_ values of 2.36 and 2.73 µM against COX1 and COX2, respectively. In general, the synthesized library has moderate activity against both enzymes (i.e., COX1 and COX2) and ortho halogenated compounds were more potent than the meta ones.
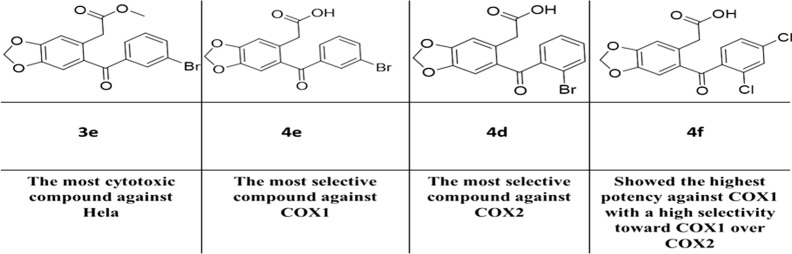

## Introduction

Some of the most used analgesics are non-steroidal anti-inflammatory drugs (NSAIDs) that target the cyclooxygenase (COX) enzymes. NSAIDs are used for various therapeutic purposes globally. Due to their wide pharmacological effects, including analgesic, anti-inflammatory and antipyretic effects, they are investigated as being some of the best choices for treating different diseases like arthritis and rheumatism, and they are widely used as analgesics. Actually, acetyl salicylic acid (ASA), one of the members of this family, has been used for more than a 100 years [1, 2]. The biosynthesis of prostaglandin H2 from arachidonic acid is catalysed by COX enzymes [3]. Prostaglandin H2 is the main component in the formation of other prostaglandins, such as thromboxane and prostacyclin, which play important roles in different biological responses [4, 5]. In fact, COX1 and COX2 are the two major isoforms of COX membrane-bound enzymes [6]. COX1 is involved in the biosynthesis of important prostaglandins which maintain the constant functions in the body, essentially in the cardiovascular and gastrointestinal systems [7]. Moreover, COX2 is an enzyme catalyst that is overexpressed in several pathophysiological events such as hyperalgesia, inflammation, and cancer [8, 9]. The structures of COX1 and COX2 enzymes are 67% identical in amino acid chains. The main difference between the two enzymes is the presence of isoleucine (Ilu523) in COX1 instead of valine (Val523) in COX2. This allows 25% greater available space in the binding region of COX2 in comparison to COX1 [10]. All of these data encourage the researchers to focus their efforts to the find COX2 selective inhibitors in order to improve treatment efficacy and to reduce the side effects that are associated with the use of non-selective inhibitors of these enzymes [11–13].

COX2 enzyme is associated with carcinogenesis and inflammatory diseases. It is suspected to induce tissue invasion of tumours, angiogenesis, and resistance to apoptosis. Moreover, COX2 plays an important role in the innate and adaptive immune response, and it contributes to immune evasion and resistance to cancer immunotherapy. However, COX inhibitors can facilitate a benefit to patients from addition of COX inhibitors when compared to standard chemotherapy [14].

**Fig. 1:**
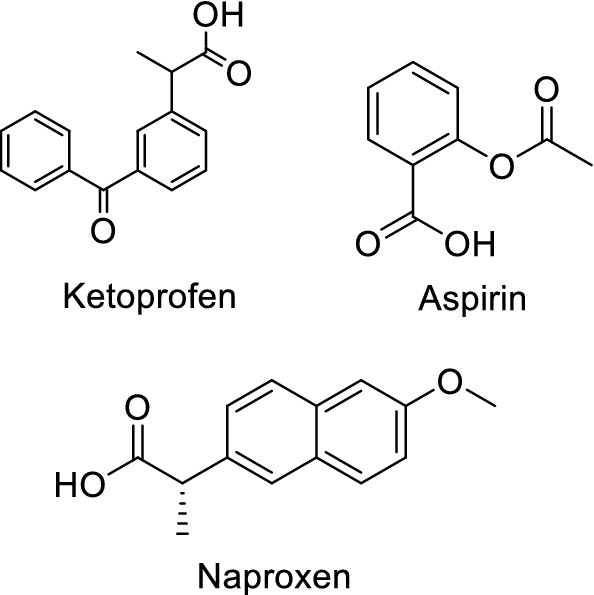
Classical NSAIDs with COOH functional group

A large number of agents with different structural features were produced in the discovery efforts of new COX2 selective inhibitors. A lot of classical non-selective NSAIDs were synthesized, approved, and used broadly, such as Ibuprofen, Naproxen, and Ketoprofen (Fig. 1), but their selectivity is too low against COX2/COX1 [15], and the previous studies were implemented to synthesize more selective agents as COX2 inhibitors by using different methods and structures [16].

According to the World Health Organization (WHO) surveys, cancer is one of the leading causes of death around the globe, and it was responsible for about 10 million deaths in 2018 [17, 18]. Around 1 in 6 people died from cancer, which is considered the largest cause of death. This is a considerably alarming estimate. WHO has recognized that 1.16 trillion US dollars were spent on the prevention and treatment of cancer in 2010 alone, and that number has increased dramatically over the years [17]. These important statistics are the result of erratic human behaviours such as smoking, which is associated with lung cancer, fruits and vegetables contaminated with pesticides and phyto-growth hormones, and the unhealthy lifestyles of modern people as well as some physical carcinogens such as radiation, some chronic diseases such as diabetes, and some infectious illnesses such Hepatitis B and C viral infections [19].

**Fig. 2:**
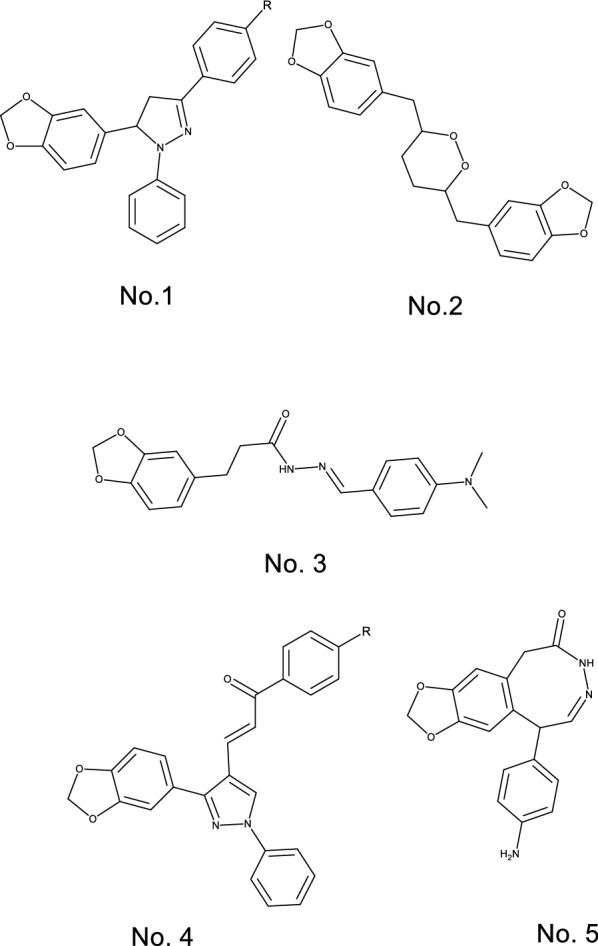
Structures of benzodioxol derivatives having various biological activities

**Fig. 3:**
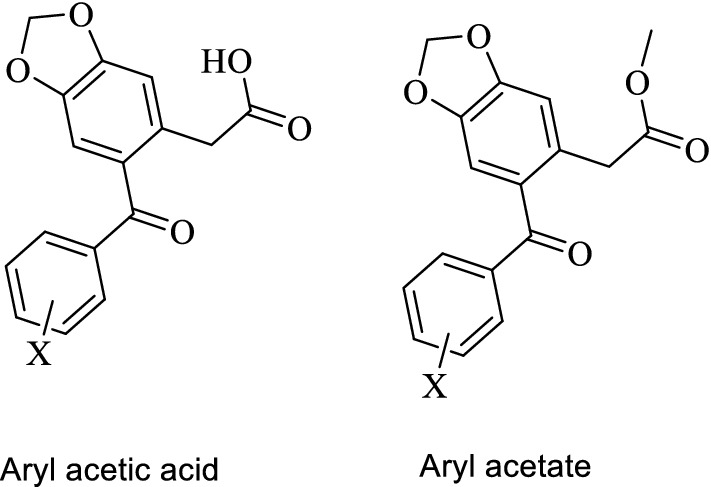
Halogenated Ketoprofen analogues as aryl acetic acid and aryl acetate

The heterocycle-containing agents have several pharmacological effects including anticancer [20, 21], anti-inflammatory [22], antioxidant [23] and analgesic effects [24]. Therefore, the Benzodioxole containing compounds (Fig. 2) have different biological activities such as anticancer, anti-tuberculosis, anti-microbial, anti-epileptic, and analgesic activity [25–30]. Various tricyclic compounds and Ketoprofen like structures were synthesized and evaluated as COX enzyme inhibitors [31, 32]. The current work aims to synthesize new compounds with a Benzodioxole core structure in two final product groups with different halogen atoms and aryl acetate and aryl acetic acid (Fig. 3), to evaluate their COX1 and COX2 inhibitory activity and to evaluate the synthesized compounds’ cytotoxic effects.

## Results and discussion

### Chemistry

**Scheme 1:**
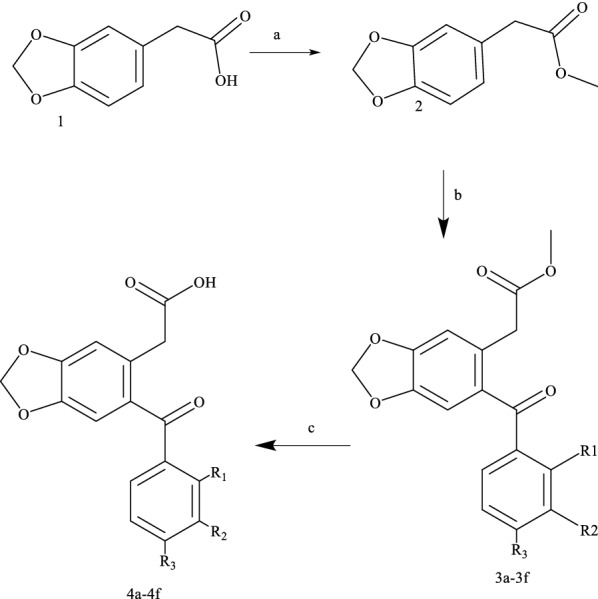
The reaction steps **a** methanol, oxalyl chloride **b** DCM, P_2_O_5_, aryl-carboxylic acid, **c** MeOH/THF/H_2_O, NaOH reflux

The Benzodioxole aryl acetate derivatives (3a-3f) and acetic acid derivatives (**4a**–**4f**) were synthesized as outlined in Scheme 1. The methyl 3,4-(methylenedioxy) phenylacetate (2) was generated by an esterification reaction of 3,4-(methylenedioxy) phenylacetic acid (1). To produce the ester (2), oxalyl chloride was added dropwise to methanol solvent and stirred for half an hour in an ice bath [33, 34]. The IR spectra of the ester (2) showed the disappearance of the broad band that belonged to the acetic acid group of (1). The aryl acetate **3a**–**3f** compounds were synthesized by dissolving the ester (**2**) in dichloromethane with benzoic acid derivatives in the presence of an excess of phosphorus pentoxide and stirring at room temperature for approximately 18 h. The ^1^H-NMR spectrum data of these compounds showed 5–7 protons (depend on the Halogen atoms for each compound) in the aromatic area, 2 protons around 6.13 ppm singlet peaks for O–C**H**_**2**_–O of benzodioxole and 5 protons were observed in area 3.40 and 3.80 ppm for –C**H**_**2**_–CO–C**H**_**3**_. According to the ^13^C-NMR spectrum, C signal of carbonyl groups was found around 195 and 171 ppm, and at 37–51 ppm two signals of aliphatic carbon were observed. The Benzodioxole acetic acid derivatives (**4a**–**4f**) were synthesized by hydrolysis reaction of the ester compounds **3a**–**3f** using NaOH [35] (see Scheme 1). The ^1^H-NMR spectrum data showed one proton with singlet peak around 12 ppm (–COOH), 2 protons around 6.13 ppm singlet peaks for O-C**H**_**2**_-O of benzodioxole and 2 protons were observed in area 3.40–3.78 ppm for –C**H**_**2**_–COOH. However, ^13^C-NMR spectrum data showed C signal of carbonyl groups around 197 and 172 ppm.

### Cyclooxygenase inhibition activity

The synthesized compounds have a structure that is similar to Ketoprofen, and because of that Ketoprofen was used as a positive control in the COX inhibition analysis of the synthesized library. All Benzodioxole acetate structures with halogens (Br, Cl, I; **3b**–**3f**) on the phenyl ring showed better activity against COX1 (IC_50_ 1.12–27.06 µM) than acetic acid Benzodioxole with halogens (IC_50_ 4.25–33.7 µM; **4b**–**4e**), except **4f** which showed the most potent inhibitory activity (IC_50_ = 0.725 µM) against the COX1 enzyme. However, the acetic acid Benzodioxole compound without a halogen (4a) showed stronger inhibition activity toward cyclooxygenase enzymes COX1 and COX2 (1.45 and 3.34 µM, respectively) than acetate Benzodioxole without a halogen compound (**3a**) toward COX1 and COX2 (12.32 and 14.34 µM, respectively). However, all Benzodioxole acetate structures with halogens (**3b**–**3f**) showed better activity against COX2 (IC_50_ 1.30–37.45 µM) than acetic acid Benzodioxole with halogens (IC_50_ 2.35–39.14 µM; **4b**–**4f**) as presented in Table 1.

**Table 1:** IC_50_ inhibition of COX1 and COX2, Selectivity ratio for COX1/COX2, and the CC_50_ on HeLa cancer cell line of the synthesized compounds

					The IC_50_ in µM of COX 1 and COX2		COX1/COX2 Ratio	HeLa Cell CC_50_ in milli-molar
Codes	X	R1	R2	R3	COX1	COX2	Selectivity	CC_50_
3a	O-CH3	H	H	H	12.320	14.340	0.859	1.49
3b	O-CH3	I	H	H	1.120	1.300	0.862	0.228
3c	O-CH3	H	I	H	27.060	37.450	0.723	1.79
3d	O-CH3	Br	H	H	1.3	1.45	0.897	1.61
3e	O-CH3	H	Br	H	2.360	2.730	0.864	0.219
3f	O-CH3	Cl	H	Cl	5.180	4.100	1.263	0.949
4a	OH	H	H	H	1.450	3.340	0.434	1.94
4b	OH	I	H	H	7.670	30.700	0.250	0.697
4c	OH	H	I	H	33.700	39.140	0.861	1.049
4d	OH	Br	H	H	4.250	2.350	1.809	0.547
4e	OH	H	Br	H	7.110	49.300	0.144	0.437
4f	OH	Cl	H	Cl	0.725	4.290	0.169	1.019
Ketoprofen					0.031	0.158	0.196	

P-values for the experiments p < 0.05

### Cytotoxic evaluation

An MTS assay was used to determine the cytotoxic effect of Benzodioxole derivatives on HeLa (cervical carcinoma cells). As shown in Table 1, four different concentrations were used (2, 1, 0.5, and 0.1 mM) to investigate the cytotoxicity of the compounds. Actually, all compounds showed inhibition of cell growth at relatively high concentrations in comparison to the IC_50_ of COX enzyme. The CC_50_ were in the range between 0.219 and 1.79 mM. The most cytotoxic compound was **3e** with a CC_50_ value of 219 µM.

### SAR study

All ortho halogenated compounds **3b**, **3d**, **4b**, and **4d** showed better activity with lower IC_50_ values than their meta halogenated compounds **3c, 3e, 4c** and **4d.** For example the IC_50_ values of compound **3b** (ortho-halogenated) against both COX1 and COX2 were 1.120 and 1.300 µM in comparison with **3d** (meta-halogenated) which were 27.060 and 37.450 µM, respectively. This depended on the theory that the ortho-halogenated compounds can make the second aromatic ring non-coplanar with the first aromatic ring, which is ideal for the COX inhibitory activity. All ester-mono halogenated compounds (ortho or meta; **3b**, **3c**, **3d** & **3e**) have better COX inhibitory activity than acetic acid mono-halogenated compounds (ortho or meta; **4b**, **4c**, **4d** & **4e**). Except for compound **4b**, all other ortho-halogenated compounds (**3b**, **3d**, and **4d)** showed better selectivity ratios (COX1/COX2) than meta-halogenated compounds. The most potent compound against COX1 enzyme was the acetic acid di-halogenated (2,4-dichloro) compound **4f**. The ortho-iodo ester compound **3b** was potent against COX2 enzyme with a good selectivity ratio (0.862).

There is no clear relationship between the ortho versus meta halogen and the cytotoxicity results. Generally, the halogenated compounds are more cytotoxic than non-halogenated (**3a** & **4a**). The most cytotoxic compound was compound **3e** (ester with Br on meta position; CC_50_ = 0.219 mM). It was more toxic than compound **3d** (ester with Br on ortho), and the same relation was found between **4e** and **4d**, respectively. In contrast, the ortho iodo halogenated compounds (**3b** and **4b)** were more toxic than meta iodo halogenated compounds (**3c** & **4c**).

In this study we can observe that our synthesized compounds have inhibition activity against both COX1 and COX2 enzymes better than some tricyclic compounds synthesized by other research teams. As published by Caliskan et al., one of pyrazol-3-propanoic acids derivatives was the most active compound in this series, and it showed a selectivity ratio of 0.93 and activity against COX1 and COX2 with an IC_50_ value relatively close to our results (1.5 and 1.6 µM, respectively). However, the inhibitory activity against COX1 for most of our synthesized compounds were very close to or better than their tested compound [36]. In another study by Assali et al., a series of pyrazole and triazole derivatives were synthesized, and one of their triazole derivatives was considered to be a highly selective COX2 inhibitor with a high selectivity ratio (162.5) [16]. Comparing our results with other studies, the results of this study clearly demonstrate that the synthesized agents have good inhibition activity against both COX1 and COX2 enzymes with relatively low IC_50_ values, and the COX selectivity ratio of the compounds synthesized in this study were better than approved drugs like ketoprofen or aspirin.

## Conclusion

The synthesized compounds showed moderate activity against COX1 and COX2 enzymes. However, most compounds have better COX2 inhibition selectivity compared to Ketoprofen. The results showed a promising group of compounds having a Benzodioxole moiety. They had better COX2 selectivity compared with Ketoprofen, and this may be due to the bigger moiety (Benzodioxole) in the synthesized compound in comparison with phenyl moiety in Ketoprofen. Future plans should include docking studies and synthesizing more analogues of this core structure to study the structure–activity relationship. This is required in order to improve their COX inhibitory activity and to achieve a better COX2 selectivity ratio. All compounds **3a**–**4f** showed cytotoxic activity on the HeLa cancer cell line at higher doses. However the effective doses towards COX enzyme were at least lesser 10 times greater than the cytotoxic concentrations.

## Experimental section

### Chemicals and instruments

All chemicals were purchased from Sigma-Aldrich and Alfa Aesar. Melting points were determined with an SMP-II Digital Melting Point Apparatus and are uncorrected. IR spectra were obtained using a Perkin Elmer Spectrum 400 FTIR/FTNIR spectrometer. ^1^H-NMR and ^13^C-NMR spectra were recorded in DMSO-d6 and were performed on two NMR instruments. The first was a Bruker 500 MHz-Avance III High-Performance Digital FT-NMR spectrometer at the Faculty of Science, Department of Chemistry, The University of Jordan, Jordan (it was used for the ^1^H-NMR of just one compound, **3e**). The second was a Bruker 300 MHz-Avance III High-Performance Digital FT-NMR spectrometer at the NMR facility at the Doping and Narcotics Analysis Laboratory of the faculty of pharmacy, Anadolu University, Turkey (it was used for both ^1^H-NMR and ^13^C-NMR for the other compounds). Tetramethylsilane was used as the internal standard. All chemical shifts were recorded as d (ppm). High resolution mass spectral data (HRMS) were collected using a Waters LCT Premier XE Mass Spectrometer (high sensitivity orthogonal acceleration time-of-flight instrument) using ESI (+) method (The instrument was coupled to an AQUITY Ultra Performance Liquid Chromatography system (Waters Corporation, Milford, MA, USA) at the Pharmacy Faculty Gazi University Ankara-Turkey. The silica gel used for the flash chromatography column had a pore size of 60 Å and 230–400 mesh particle size, 40–63 μm particle size. The inhibitory activity of ovine COX1 and human recombinant COX2 enzymes was determined using a COX inhibitor screening assay kit No. 560131 (Cayman Chemical, USA). The yellow product of this enzymatic reaction was determined using a UV spectrophotometer with a Microplate Reader (BioRad, Japan) at a wavelength of 415 nm. HeLa Cervical Carcinoma cell line was purchased from ATCC (ATCC^®^ CCL-2™), and the cyototoxicty test of the cell viability was assessed by the CellTilter 96^®^ Aqueous One Solution Cell Proliferation (MTS) assay according to the manufacturer’s instructions (Promega Corporation, Madison, WI) (Additional file 1).

### Chemistry method

#### Synthesis of methyl 2-(2*H*-1,3-benzodioxol-5-yl) acetate synthesis **2**

The 3,4-(methylenedioxy)phenylacetic acid (1) (8 g, 44.40 mmol) was dissolved in methanol, then it was cooled in an ice bath to 0 °C. Then oxalyl chloride (4 mL, 46.80 mmol) was added dropwise, and the reaction mixture was stirred for 30–45 min. The reaction mixture was then evaporated under vacuum and the resulting residue was diluted with ethyl acetate solvent and washed with saturated sodium bicarbonate (NaHCO_3_) and distilled water, sequentially. The organic layer was dried with sodium sulphate, then filtered and evaporated again to concentrate it. In the last step, it was purified by silica gel column chromatography by using a hexane:ethyl acetate solvent system (50%:50%). The resulting compound (2) was a yellow oil with 94% yield.

#### General synthesis procedure for ketoester (**3a**–**3f**) derivatives

The benzoic acid derivatives (1.46 g, 6.68 mmol) and phosphorus pentoxide (5 g) were added to a stirred solution of dichloromethane (60 mL) and compound (2) (1 g, 5.14 mmol). Then, the mixture was stirred at room temperature for 18 h before distilled water (60 mL) was cautiously added, and the mixture was extracted with ethyl acetate twice (60 mL). Then, the organic layer was separated and treated with 1 M NaOH (60 mL), brine (60 mL), and twice with 60 mL of distilled water. The organic layer was dried with sodium sulphate, filtered, evaporated under vacuum, and then purified by silica gel column chromatography with different solvent systems.

##### Methyl 2-(6-benzoylbenzo[d][1,3]dioxol-5-yl)acetate (3a)

Purified by silica gel column chromatography using *n*-hexane: ethyl acetate solvent system (3:2). Crude yellow semi solid, Yield 75%; ESI–MS: 299.0919 (100), 300 (20), 301 (2), For C_17_H_15_O_5_. IR (FTIR/FTNIR-ATR): 1737 cm^−1^ ester carbonyl (C=O), 1661 cm^−1^ keton carbonyl (C=O). ^1^H NMR (DMSO-d_6_, 300 MHz) δ ppm: 7.62–7.67 (3H, m, Ar–H), 7.52 (2H, t, *J* = 7.8 Hz, Ar–H), 7.05 (1H, s, Ar–H), 6.89 (1H, s, Ar–H), 6.12 (2H, s, O–CH_2_–O), 3.74 (2H, s, –CH_2_–C=O), 3.47 (3H, s, O–CH_3_). ^13^C-NMR (DMSO-d_6_, 75 MHz) δ ppm: 196.53, 171.61, 149.66, 146.04, 138.08, 133.39, 131.59, 130.21, 130.00, 129.73, 129.44, 128.95, 112.66, 110.34, 102.45, 51.89, and 38.36.

##### Methyl 2-(6-(2-iodobenzoyl)benzo[d][1,3]dioxol-5-yl)acetate (3b)

Purified by silica gel column chromatography using *n*-hexane: ethyl acetate solvent system (1:1). Semi solid product, Yield 90%. ESI–MS: 424.9875 (100), 425.99 (20), for C_17_H_14_IO_5_. IR (FTIR/FTNIR-ATR): 1740 cm^−1^ ester carbonyl (C = O), 1659 cm^−1^ keton carbonyl (C=O). ^1^H NMR (DMSO-d_6_, 300 MHz) δ ppm: 7.95 (1H, d, *J* = 7 Hz, Ar–H), 7.51 (1H, t, *J* = 7.5 Hz, Ar–H), 7.23–7.30 (2H, m, Ar–H), 7.11 (1H, s, Ar–H), 6.64 (1H, s, Ar–H), 6.13 (2H, s, O-CH_2_-O), 3.92 (2H, s, –CH_2_–C=O), 3.59 (3H, s, O–CH_3_). ^13^C-NMR (DMSO-d_6_, 75 MHz) δ ppm: 197.19, 171.49, 151.23, 146.51, 145.12, 139.78, 134.05, 133.30, 131.97, 128.95, 128.60, 113.60, 112.03, 102.93, 93.46, 51.98.

##### Methyl 2-(6-(4-iodobenzoyl)benzo[d][1,3]dioxol-5-yl)acetate (3c)

Purified by silica gel column chromatography using *n*-hexane: ethyl acetate solvent system (1:1). Powder product mp: 119–121 °C, Yield 87%. ESI–MS: 424.96 (100), 425.99 (20), for C_17_H_14_IO_5_. IR (FTIR/FTNIR-ATR): 1735 cm^−1^ ester carbonyl (C=O), 1660 cm^−1^ keton carbonyl (C=O). ^1^H NMR (DMSO-d_6_, 300 MHz) δ ppm: 7.92 (2H, d, *J* = 8.4 Hz, Ar–H), 7.41 (2H, d, *J* = 8.8 Hz, Ar–H), 7.06 (1H, s, Ar–H), 6.92 (1H, s, Ar–H), 6.13 (2H, s, O–CH_2_–O), 3.75 (2H, s, –CH_2_–C=O), 3.47 (3H, s, O–CH_3_). ^13^C-NMR (DMSO-d_6_, 75 MHz) δ ppm: 195.94, 171.61, 149.79, 138.52, 137.89, 137.40, 131.94, 131.20, 130.14, 112.70, 110.38, 102.49, 102.05, 52.02, 38.29.

##### Methyl 2-(6-(2-bromobenzoyl)benzo[d][1,3]dioxol-5-yl)acetate (3d)

Purified by silica gel column chromatography using *n*-hexane: ethyl acetate solvent system (4:1). Powder product, mp: 85–87 °C, Yield 85%; ESI–MS: 377.00 (100), 379 (98), 380 (20), For C_17_H_14_BrO_5_. IR (FTIR/FTNIR-ATR): 1740 cm^−1^ ester carbonyl (C=O), 1658 cm^−1^ keton carbonyl (C=O). ^1^H NMR (DMSO-d_6_, 300 MHz) δ ppm: 7.34–7.77 (4H, m, Ar–H), 7.11 (1H, s, Ar–H), 6.69 (1H, s, Ar–H), 6.14 (2H, s, O–CH_2_–O), 3.93 (2H, s, –CH_2_–C=O), 3.59 (3H, s, O–CH_3_). ^13^C-NMR (DMSO-d_6_, 75 MHz) δ ppm: 196.53, 171.61, 149.66, 146.04, 138.08, 133.39, 131.59, 130.21, 130.00, 129.73, 129.44, 128.95, 112.66, 110.34, 102.45, 51.89, 38.36.

##### Methyl 2-(6-(3-bromobenzoyl)benzo[d][1, 3]dioxol-5-yl)acetate (3e)

Purified by silica gel column chromatography using *n*-hexane: ethyl acetate solvent system (3:2). Powder product, mp: 72.5–74.5 °C, Yield 79%; ESI–MS: 377.00 (100), 379 (98), 380 (20), for C_17_H_14_BrO_5_. IR (FTIR/FTNIR-ATR): 1742 cm^−1^ ester carbonyl (C=O), 1655 cm^−1^ keton carbonyl (C=O). ^1^H NMR (DMSO-d_6_, 500 MHz) δ ppm: 7.86 (1H, d, *J* = 8 Hz, Ar–H), 7.77 (1H, s, Ar–H), 7.64 (1H, d, *J* = 8 Hz, Ar–H), 7.50 (1H, t, *J* = 8 Hz, Ar–H), 7.06 (1H, s, Ar–H), 6.95 (1H, s, Ar–H), 6.15 (2H, s, O–CH_2_–O), 3.77 (2H, s, –CH_2_–C=O), 3.49 (3H, s, O–CH_3_).

##### Methyl 2-(6-(2,4-dichlorobenzoyl)benzo[d][1, 3]dioxol-5-yl)acetate (3f)

Purified by silica gel column chromatography using *n*-hexane: ethyl acetate solvent system (1:1). Powder product, mp: 95–97 °C, Yield 83%; ESI–MS: 367.01 (100), 369 (67), for C_17_H_13_Cl_2_O_5_. IR (FTIR/FTNIR-ATR): 1760 cm^−1^ ester carbonyl (C=O), 1633 cm^−1^ keton carbonyl (C=O). ^1^H NMR (DMSO-d_6_, 300 MHz) δ ppm: 7.79–7.82 (2H, m, Ar–H), 7.59 (1H, d, *J* = 8.4 Hz, Ar–H), 7.07 (1H, s, Ar–H), 7.00 (1H, s, Ar–H), 6.14 (2H, s, O–CH_2_–O), 3.78 (2H, s, –CH_2_–C=O), 3.48 (3H, s, O-CH_3_). ^13^C-NMR (DMSO-d_6_, 75 MHz) δ ppm: 194.29, 171.68, 150.13, 146.17, 138.56, 136.07, 131.70, 131.34, 130.62, 130.52, 130.33, 116.68, 112.82, 110.64, 102.06, 52.05, 38.33.

##### General synthesis procedure of 2-(6-benzoyl-2H-1,3-benzodioxol-5-yl)acetic acid (**4a**–**4f**)

The ketoesters **3a**–**3f** (450 mg, 1.35 mmol) were dissolved in methanol/H_2_O/THF (12/12/12 mL), then NaOH (540.9 mg, 13.5 mmol) was added. The solution was heated in an oil bath and refluxed for 4 h before being cooled to room temperature. The solution was then evaporated, and the residue was made acidic by adding HCl 2 N (pH = 2). The precipitate was filtered and concentrated under vacuum to give the crude products **4a**–**4f**.

##### 2-(6-benzoylbenzo[d][1,3]dioxol-5-yl)acetic acid (4a)

Purified by silica gel column chromatography using *n*-hexane: ethyl acetate solvent system (3:2). Powder product, mp: 184.5–186.5 °C, Yield 97%; ESI–MS: 285.07 (100), 286 (20), for C_16_H_13_O_5_. IR (FTIR/FTNIR-ATR): 1770 cm^−1^ acetic acid carbonyl (C=O), 1655 cm^−1^ keton carbonyl (C=O). ^1^H NMR (DMSO-d_6_, 300 MHz) δ ppm: 12.18 (1H, s, OH), 7.49–7.70 (5H, m, Ar–H), 7.03 (1H, s, Ar–H), 6.86 (1H, s, Ar–H), 6.11 (2H, s, O–CH_2_–O), 3.67 (2H, s, –CH_2_–C=O). ^13^C-NMR (DMSO-d_6_, 75 MHz) δ ppm: 96.64, 172.65, 149.48, 138.12, 133.68, 133.33, 131.73, 130.68, 130.26, 130.01, 129.20, 128.90, 112.62, 110.16, 102.32, 38.59.

##### 2-(6-(2-iodobenzoyl)benzo[d][1,3]dioxol-5-yl)acetic acid (4b)

Purified by silica gel column chromatography using *n*-hexane: ethyl acetate solvent system (3:2). Powder product, mp: 147–149 °C, Yield 92%; ESI–MS: 410.97 (100), 411 (20), for C_16_H_12_IO_5_. IR (FTIR/FTNIR-ATR): 1754 cm^−1^ acetic acid carbonyl (C=O), 1653 cm^−1^ keton carbonyl (C=O). ^1^H NMR (DMSO-d_6_, 300 MHz) δ ppm: 7.95 (1H, d, *J* = 7.8 Hz, Ar–H), 7.50 (1H, t, *J* = 7.8 Hz, Ar–H), 7.23–7.31 (2H, m, Ar–H), 7.06 (1H, s, Ar–H), 6.61 (1H, s, Ar–H), 6.11 (2H, s, O–CH_2_–O), 3.83 (2H, s, –CH_2_–C=O). ^13^C-NMR (DMSO-d_6_, 75 MHz) δ ppm: 197.14, 172.60, 150.97, 146.17, 145.23, 139.81, 134.23, 131.97, 129.31, 129.14, 128.54, 113.48, 111.77, 102.75, 93.51.

##### 2-(6-(4-iodobenzoyl)benzo[d][1,3]dioxol-5-yl)acetic acid (4c)

Purified by silica gel column chromatography using *n*-hexane: ethyl acetate solvent system (3:2). Powder product, mp: 239.5–241.5 °C, Yield 89%; ESI–MS: 410.97 (100), 411 (20), for C_16_H_12_IO_5_. IR (FTIR/FTNIR-ATR): 1760 cm^−1^ acetic acid carbonyl (C=O), 1660 cm^−1^ keton carbonyl (C=O). ^1^H NMR (DMSO-d_6_, 300 MHz) δ ppm: 12.20 (1H, s, OH), 7.91 (2H, d, *J* = 8.7 Hz, Ar–H), 7.42 (2H, d, *J* = 8.4 Hz, Ar–H), 7.03 (1H, s, Ar–H), 6.90 (1H, s, Ar–H), 6.11 (2H, s, O–CH_2_–O), 3.67 (2H, s, –CH_2_–C=O). ^13^C-NMR (DMSO-d_6_, 75 MHz) δ ppm: 196.04, 172.63, 149.60, 145.85, 138.14, 137.83, 137.44, 131.99, 131.30, 130.77, 112.63, 110.17, 102.37, 101.95, 38.56.

##### 2-(6-(2-bromobenzoyl)benzo[d][1,3]dioxol-5-yl)acetic acid (4d)

Purified by silica gel column chromatography using *n*-hexane: ethyl acetate solvent system (3:2). Powder product, mp: 145–147 °C, Yield 87%; ESI–MS: 362.99 (100), 364 (98), 365 (20) for C_16_H_12_BrO_5_. IR (FTIR/FTNIR-ATR): 1766 cm^−1^ acetic acid carbonyl (C=O), 1664 cm^−1^ keton carbonyl (C=O). ^1^H NMR (DMSO-d_6_, 300 MHz) δ ppm: 7.41–7.73 (4H, m, Ar–H), 6.96 (1H, s, Ar–H), 6.61 (1H, s, Ar–H), 6.07 (2H, s, O-CH_2_-O), 3.68 (2H, s, –CH_2_–C=O), 3.42 (1H, bs, O–H). ^13^C-NMR (DMSO-d_6_, 75 MHz) δ ppm: 195.59, 172.70, 150.48, 133.34, 132.50, 132.01, 130.31, 127.99, 119.14, 112.82, 112.45, 111.03, 105.10, 102.38, 101.57.

##### 2-(6-(3-bromobenzoyl)benzo[d][1, 3]dioxol-5-yl)acetic acid (4e)

Purified by silica gel column chromatography using *n*-hexane: ethyl acetate solvent system (3:2). Powder product, mp: 154–156 °C, Yield 96%; ESI–MS: 362.98 (100), 364 (98), 365 (20) for C_16_H_12_BrO_5_. IR (FTIR/FTNIR-ATR): 1759 cm^−1^ acetic acid carbonyl (C=O), 1658 cm^−1^ keton carbonyl (C=O). ^1^H NMR (DMSO-d_6_, 300 MHz) δ ppm: 12.24 (1H, s, O–H) 7.75–7.78 (2H, m, Ar–H), 7.64 (1H, d, *J* = 8.1 Hz, Ar–H), 7.48 (1H, t, *J* = 8.1 Hz, Ar–H), 7.04 (1H, s, Ar–H), 6.92 (1H, s, Ar–H), 6.12 (2H, s, O–CH_2_–O), 3.70 (2H, s, –CH_2_–C=O). ^13^C-NMR (DMSO-d_6_, 75 MHz) δ ppm: 195.25, 172.72, 149.80, 145.88, 140.39, 135.85, 132.47, 131.17, 131.09, 131.04, 129.34, 122.12, 112.70, 110.31, 102.44, 38.58.

##### 2-(6-(2,4-dichlorobenzoyl)benzo[d][1,3]dioxol-5-yl)acetic acid (4f)

Purified by silica gel column chromatography using *n*-hexane: ethyl acetate solvent system (1:1). Solid product, mp: 168.5–170 °C, Yield 91%; ESI–MS: 352.99 (100), 354 (67) for C_16_H_12_Cl_2_O_5_. IR (FTIR/FTNIR-ATR): 1768 cm^−1^ acetic acid carbonyl (C=O), 1657 cm^−1^ keton carbonyl (C=O). ^1^H NMR (DMSO-d_6_, 300 MHz) δ ppm: 12.25 (1H, s, OH), 7.77–7.81 (2H, m, Ar–H), 7.60 (1H, dd, *J* = 8.3, 1.8 Hz, Ar–H), 7.05 (1H, s, Ar–H), 6.98 (1H, s, Ar–H), 6.13 (2H, s, O–CH_2_–O), 3.71 (2H, s, –CH_2_–C=O). ^13^C-NMR (DMSO-d_6_, 75 MHz) δ ppm: 194.43, 172.73, 149.41, 145.94, 138.60, 135.99, 131.75, 131.29, 131.20, 130.74, 130.36, 129.84, 112.74, 110.40, 102.47, 38.56 (Additional file 1).

#### Biological COX assay method

The ability of the synthesized a series to prevent the conversion of arachidonic acid (AA) to PGH2 by human recombinant COX2 and bovine COX1 was assessed using a COX inhibitor screening assay kit (Item No: 560131) according to the Cayman chemical manufacturer’s guidelines (USA). The 50% inhibitory concentration (IC_50_) of COX1/COX2 activity of the compounds was carried out. The assay was run in duplicate with three concentrations (50, 20, and 5 µM). A standard curve of eight concentrations of prostaglandin, a non-specific binding sample, and a maximum binding sample was used, as instructed in the kit manual, to determine the inhibition of sample compound by applying the multiple regression generated best-fit line. The percentage inhibition of the three concentrations was used to calculate the IC_50_ [16].

#### Cell culture and cytotoxicity assay

HeLa Cervical Carcinoma was cultured in RPMI-1640 media and supplemented with 10% fetal bovine serum, 1% penicillin/streptomycin antibiotics and 1% l-glutamine. Cells were grown in a humidified atmosphere with 5% CO_2_ at 37 °C, and they were seeded in 2.6 × 104 cells/well in a 96-well plate. After 48 h, the cells were confluent, the media was changed, and cells were incubated with four concentrations (2, 1, 0.5, and 0.2 mM) of the tested compounds for 24 h. Cell viability was assessed by the CellTilter 96^®^ Aqueous One Solution Cell Proliferation (MTS) Assay according to the manufacturer’s instructions (Promega Corporation, Madison, WI). Briefly, at the end of the treatment, 20 μL of MTS solution per 100 μL of media was added to each well and incubated at 37 °C for 2 h. Absorbance was measured at 490 nm [37].

## Supplementary information

**Additional file 1:** The data in the addtional file include NMR spectrum files and HRMS file of all newly synthesized compounds described in this article.

## Data Availability

The datasets used and/or analysed during the current study available from the corresponding author on reasonable request.

## Abbreviations

COX: Cyclooxygenase
FTIR: Fourier-transform infrared spectroscopy
HRMS: High resolution mass spectroscopy
^1^H NMR: Proton nuclear magnetic resonance
^13^C NMR: Carbon nuclear magnetic resonance
µM: Micro molar
mM: Mili molar
NSAIDs: Non-steroidal anti-inflammatory drugs
ASA: acetyl salicylic acid
WHO: World Health Organization
HeLa: Cervical carcinoma cells
IC_50_: 50% Inhibition concentration
CC_50_: 50% Cytotoxic concentration
NaHCO_3_: Sodium bicarbonate
NaOH: Sodium hydroxide
DMSO: Dimethyl sulfoxide
AA: Arachidonic acid
