# Supplementary material for: Design, synthesis and biological evaluation of novel benzodioxole derivatives as COX inhibitors and cytotoxic agents
Source: BMC Chem. 2020 Sep 7;14(1):54. doi: 10.1186/s13065-020-00706-1 (PMC7487730; doi:10.1186/s13065-020-00706-1)

Design, Synthesis and Biological Evaluation of Novel Benzodioxole Derivatives as COX Inhibitors and Cytotoxic Agents

Mohammed Hawash^a^*, Nidal Jaradat^a^, Saba Hameedi^a^, Ahmed Mousa^b^

^a^Department of Pharmacy, Faculty of Medicine and Health Sciences, An-Najah National University, Nablus 00970, Palestine.

^b^Department of Biomedical Sciences, Faculty of Medicine and Health Sciences, An-Najah National University, Nablus 00970, Palestine.

Corresponding author:

Mohammed Hawash, P.O. Box 7, **E-mail:* [*mohawash@najah.edu*](mailto:mohawash@najah.edu)*, +972-569-939-939*

HRMS

**3b**

3a

3f

4e

4c

3c


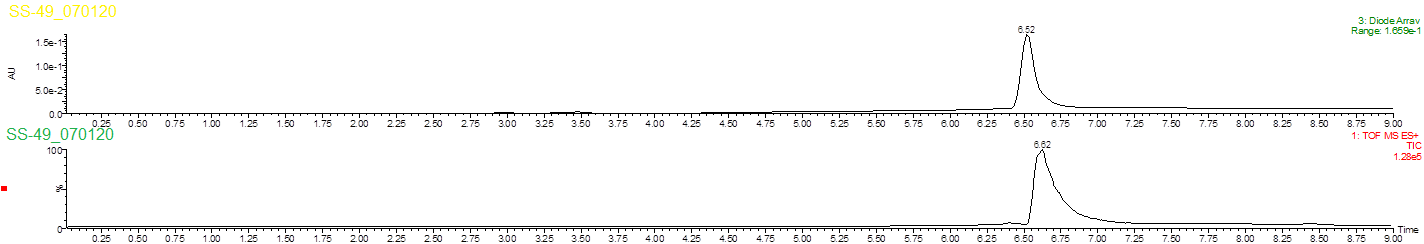


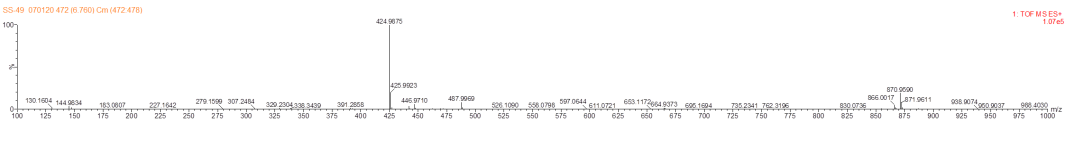


3e

3d

4f

4a

4b

3a


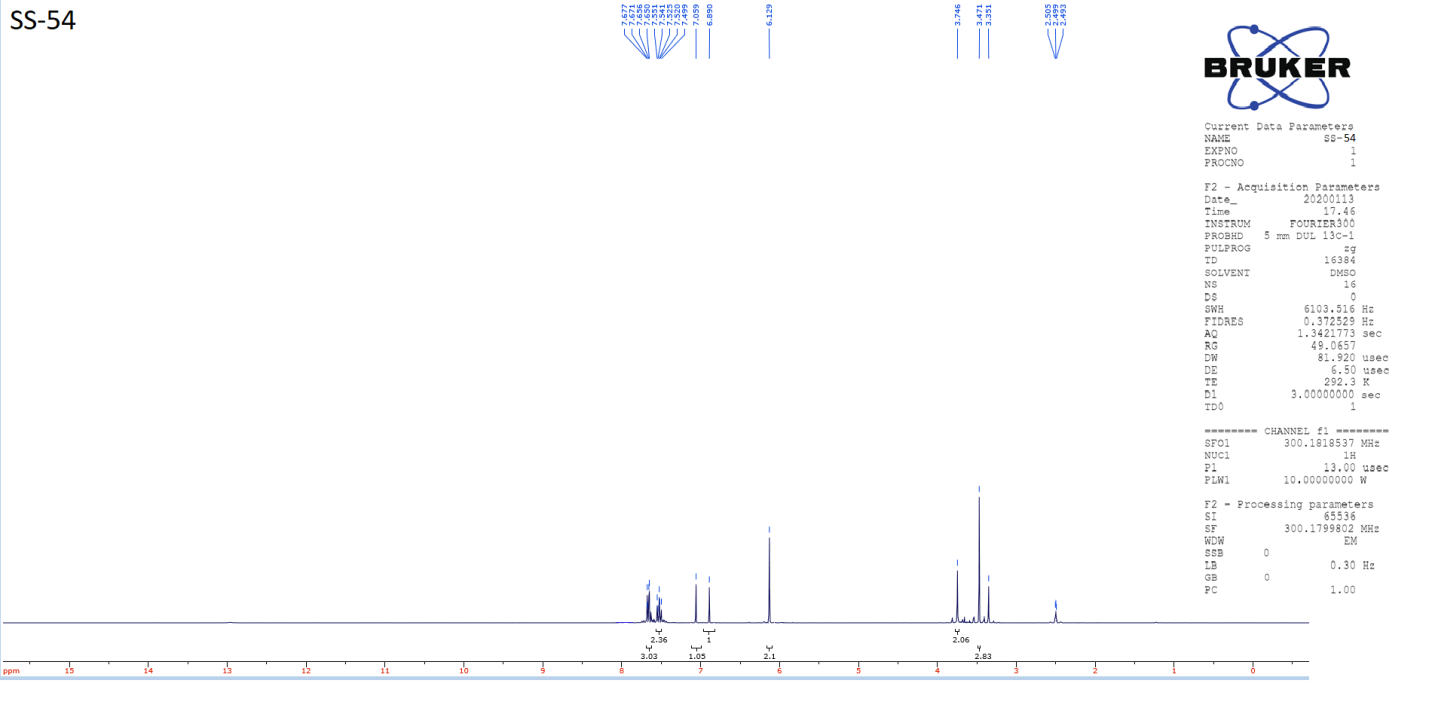


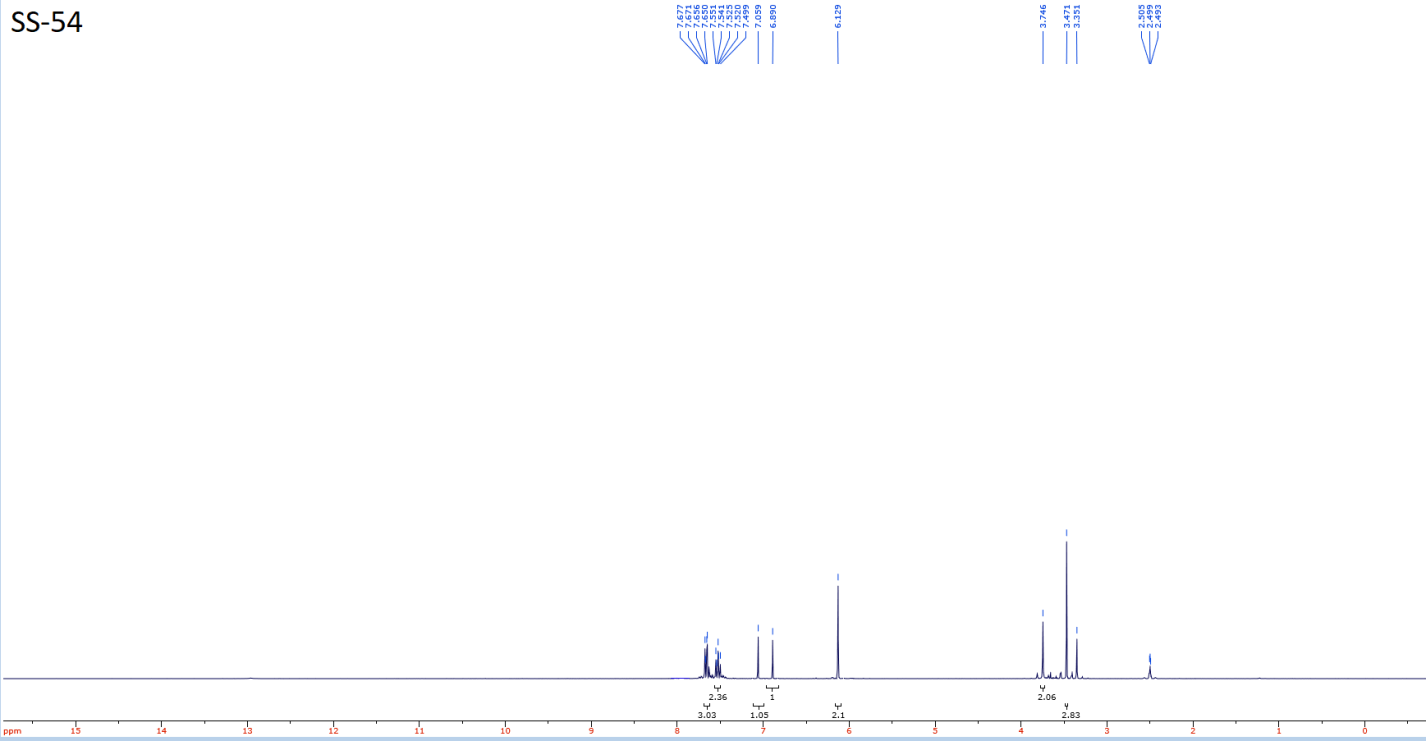


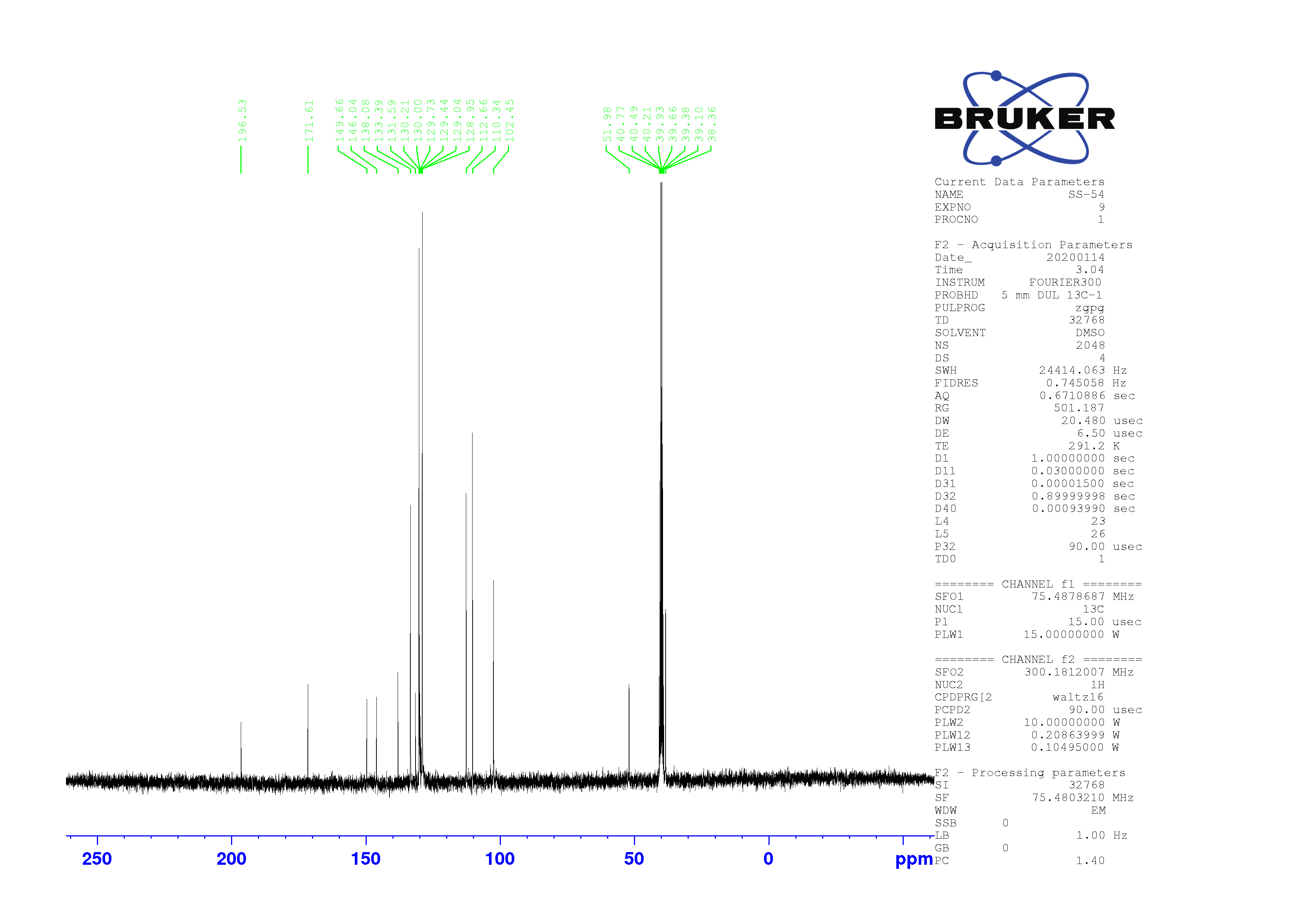


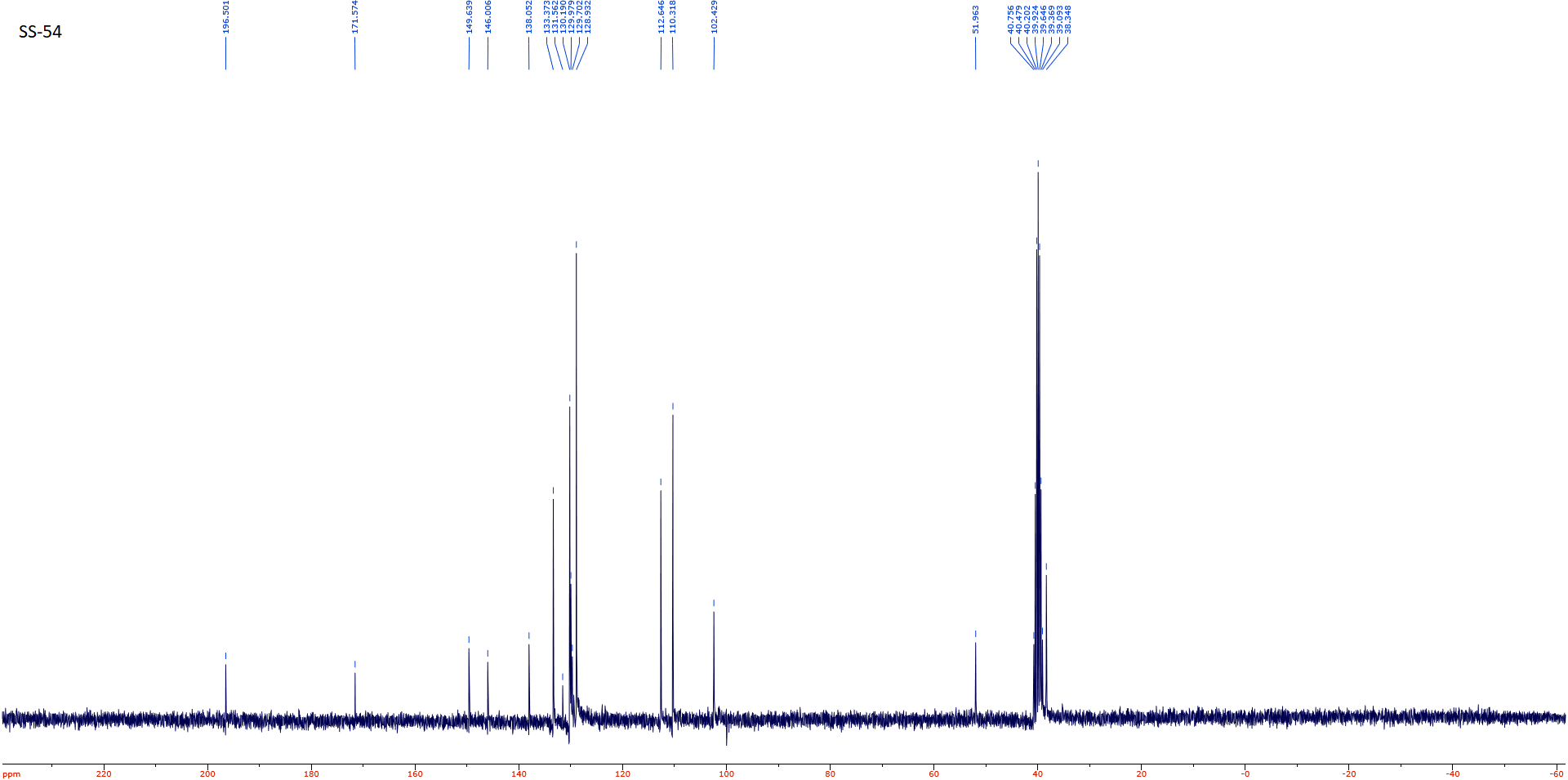


3c


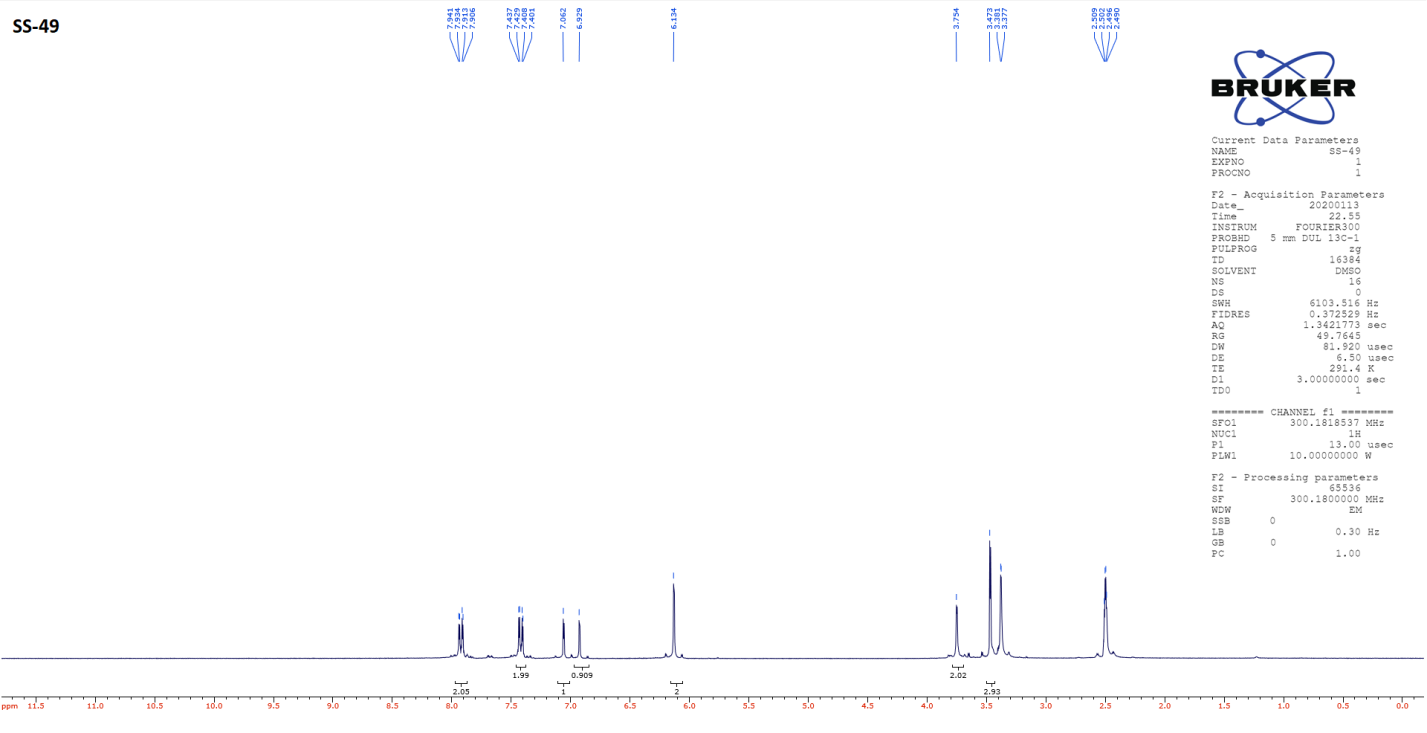


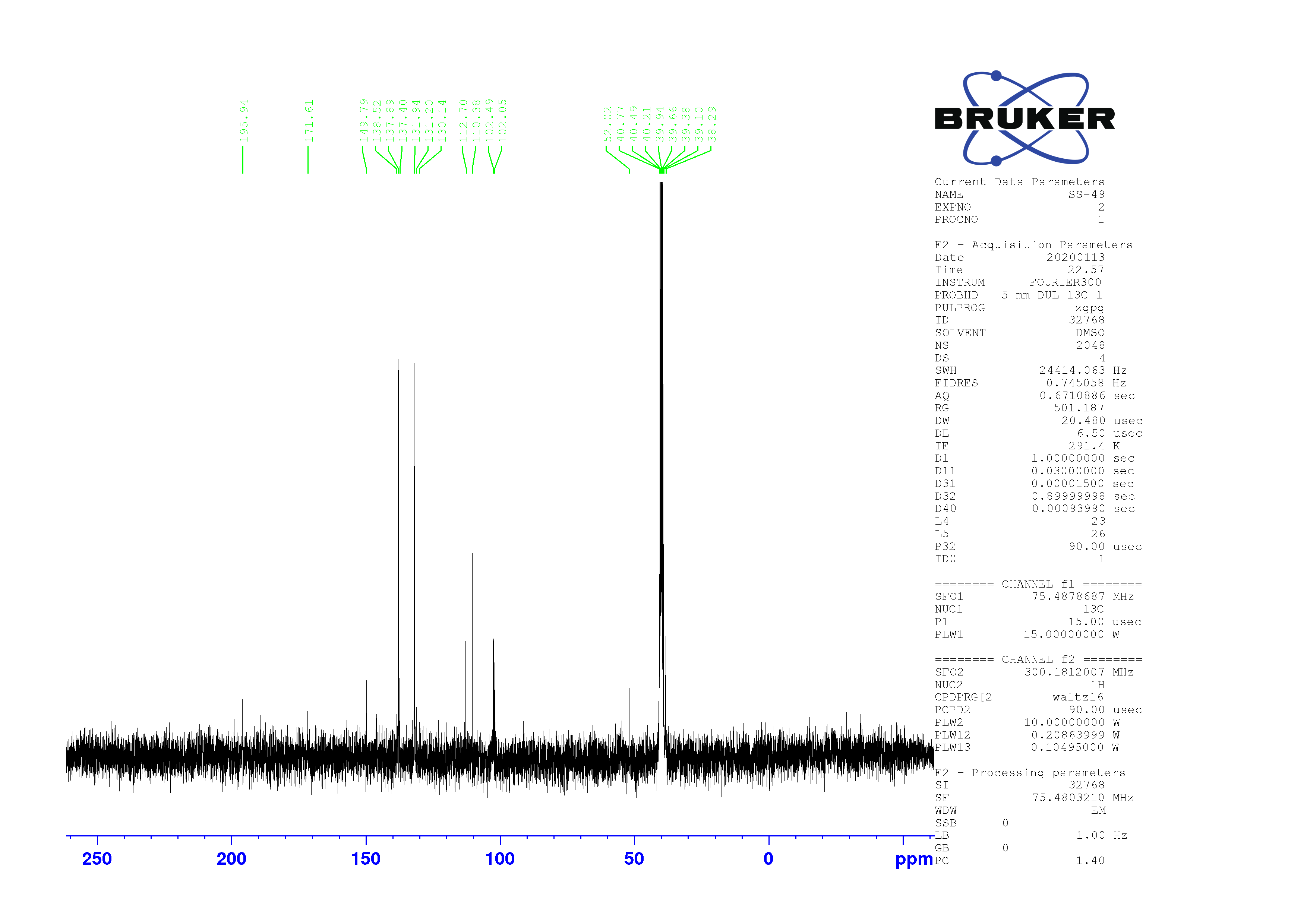


3f


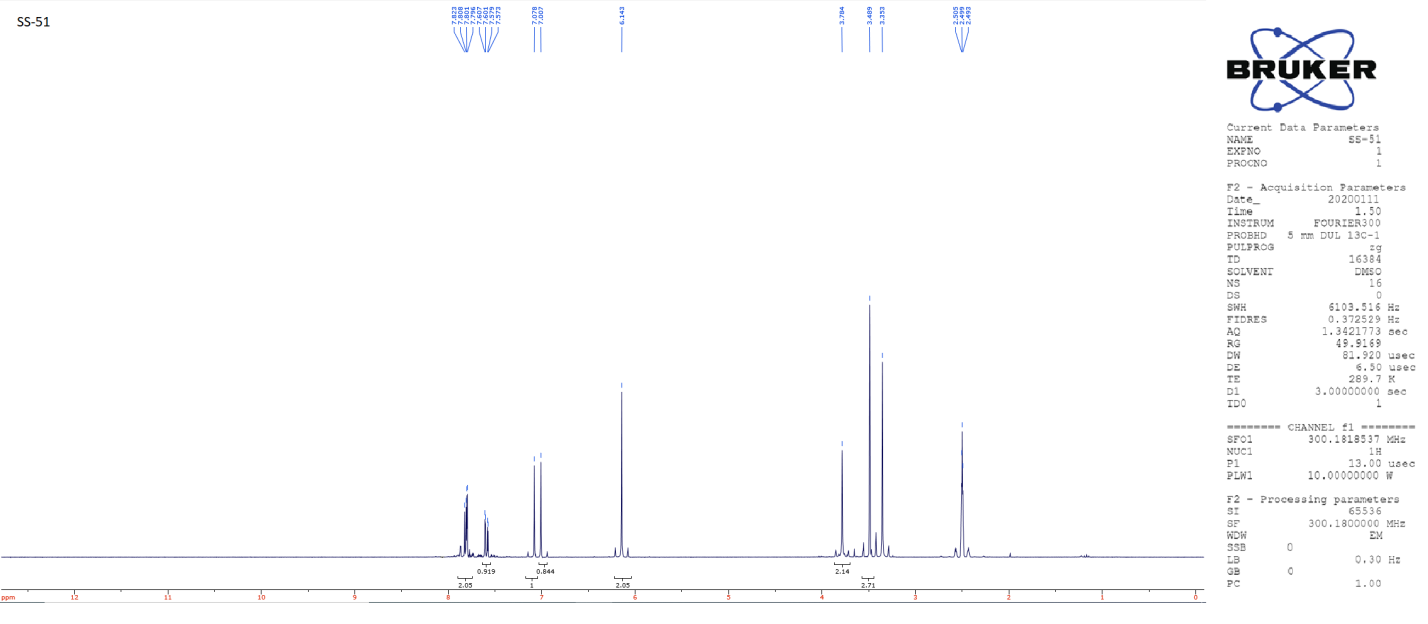


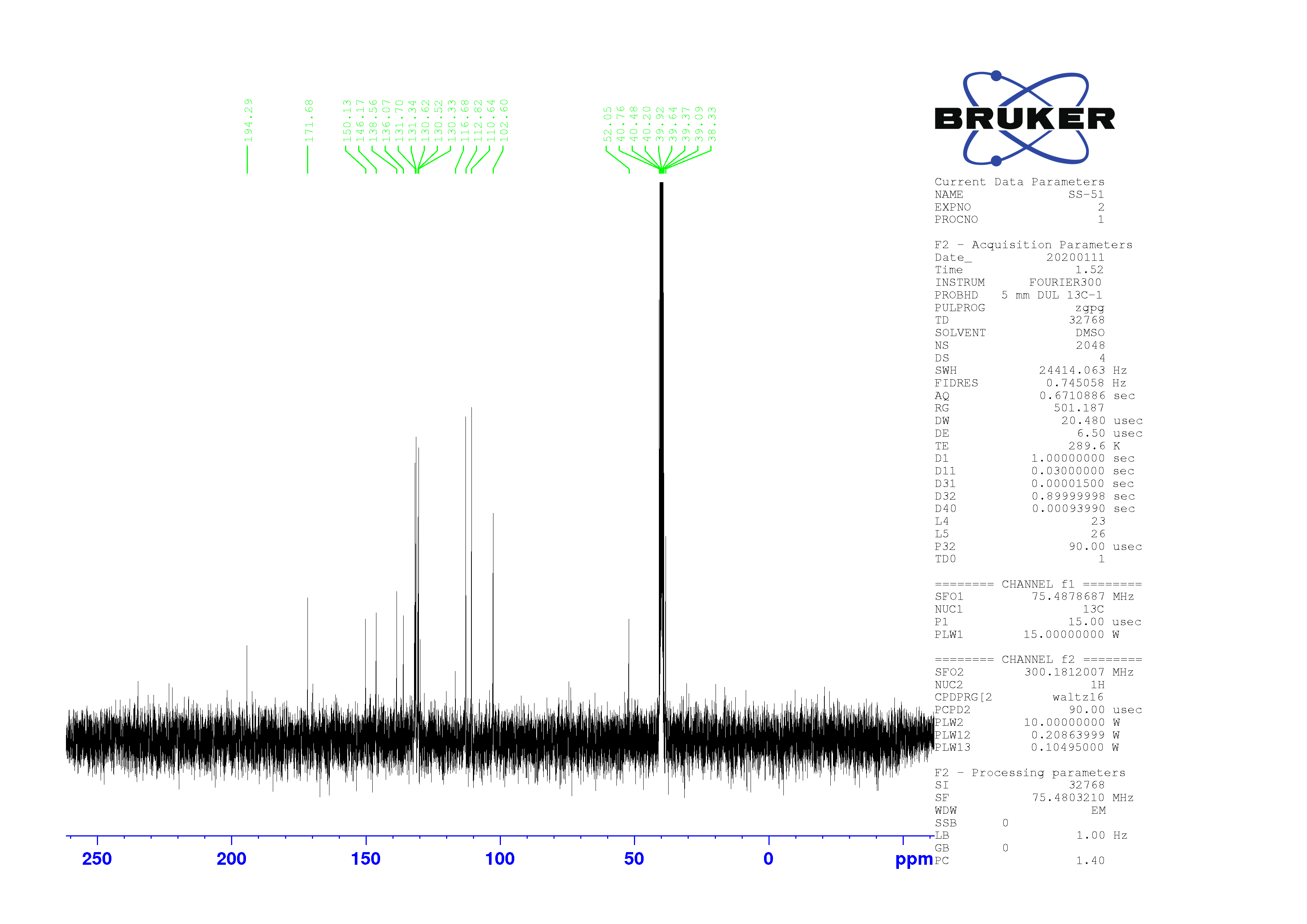


3d


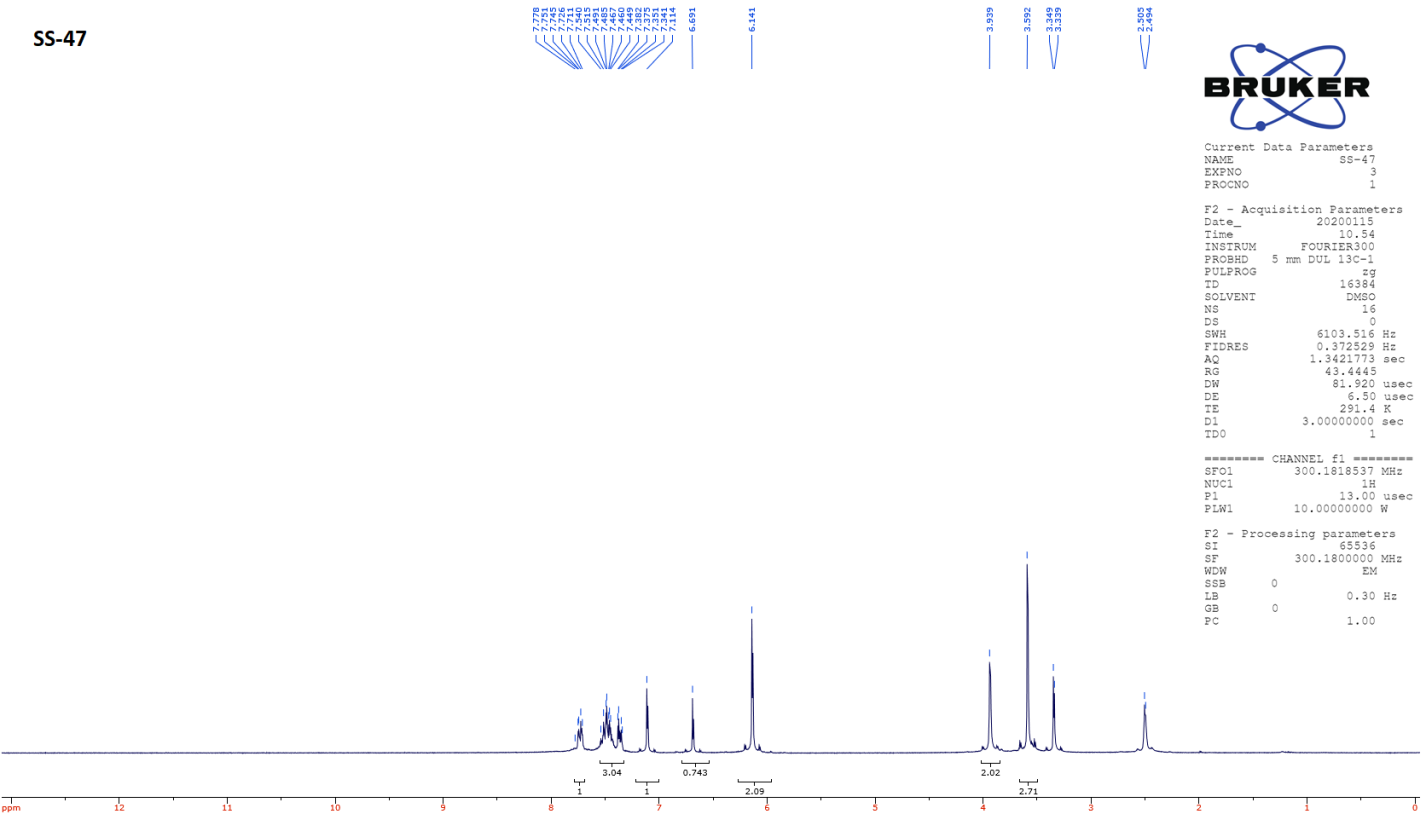


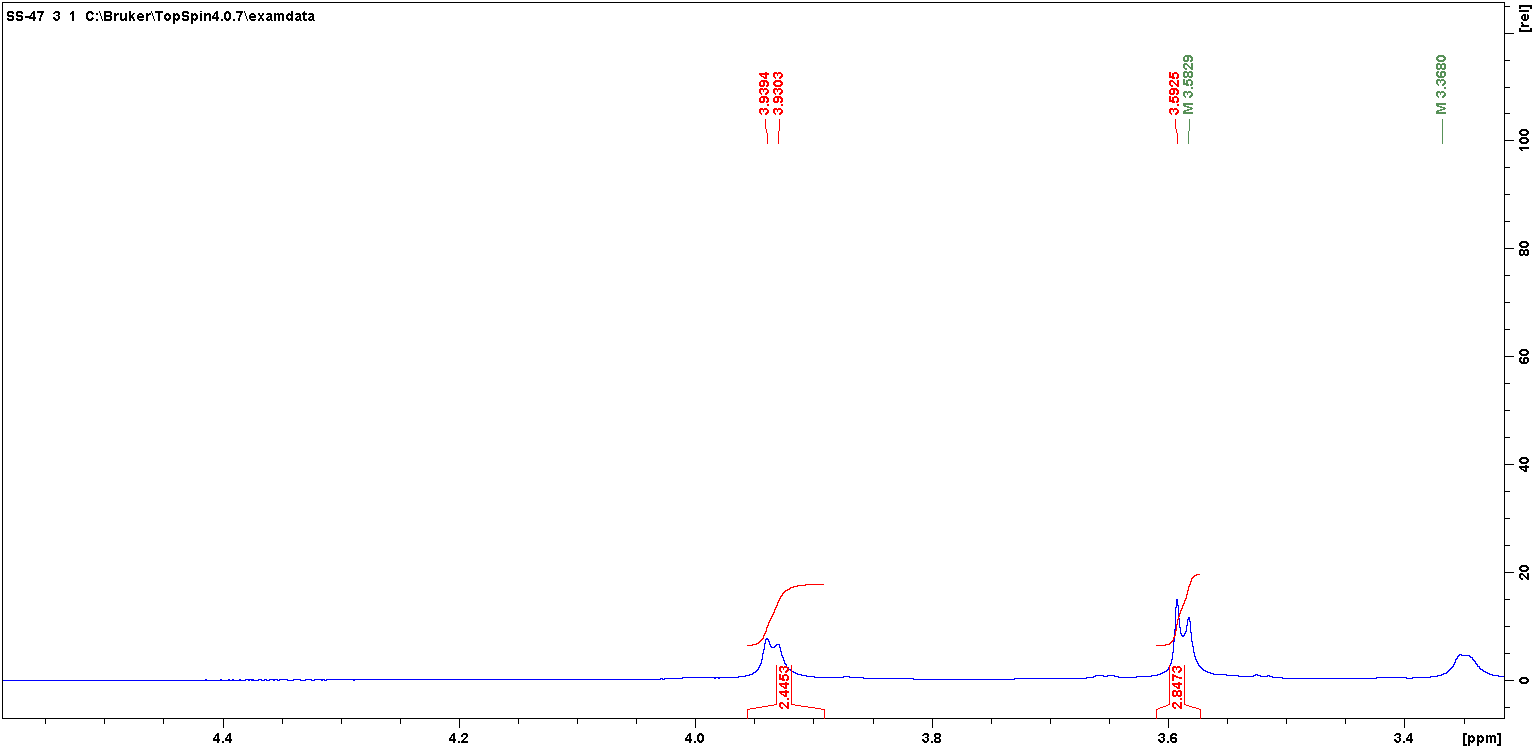


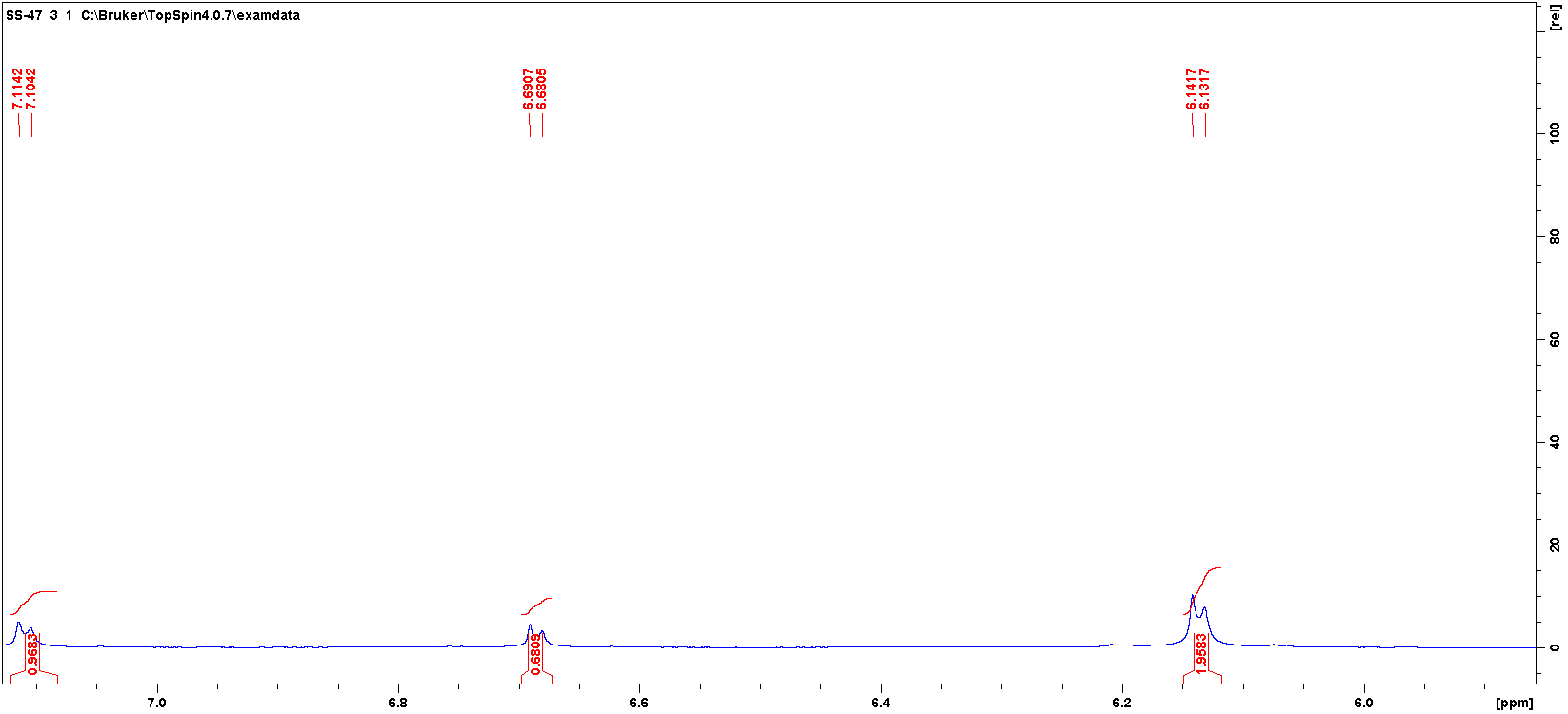


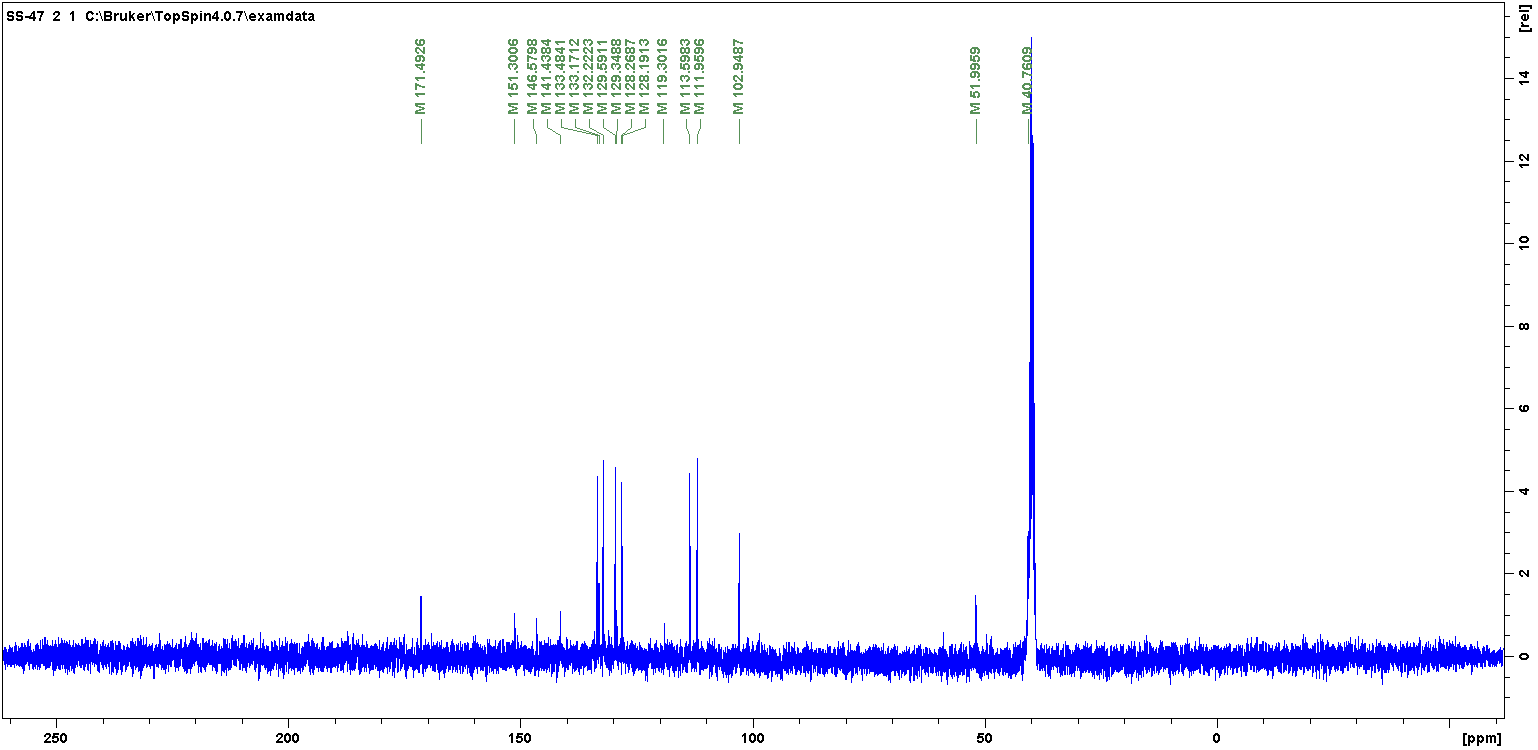


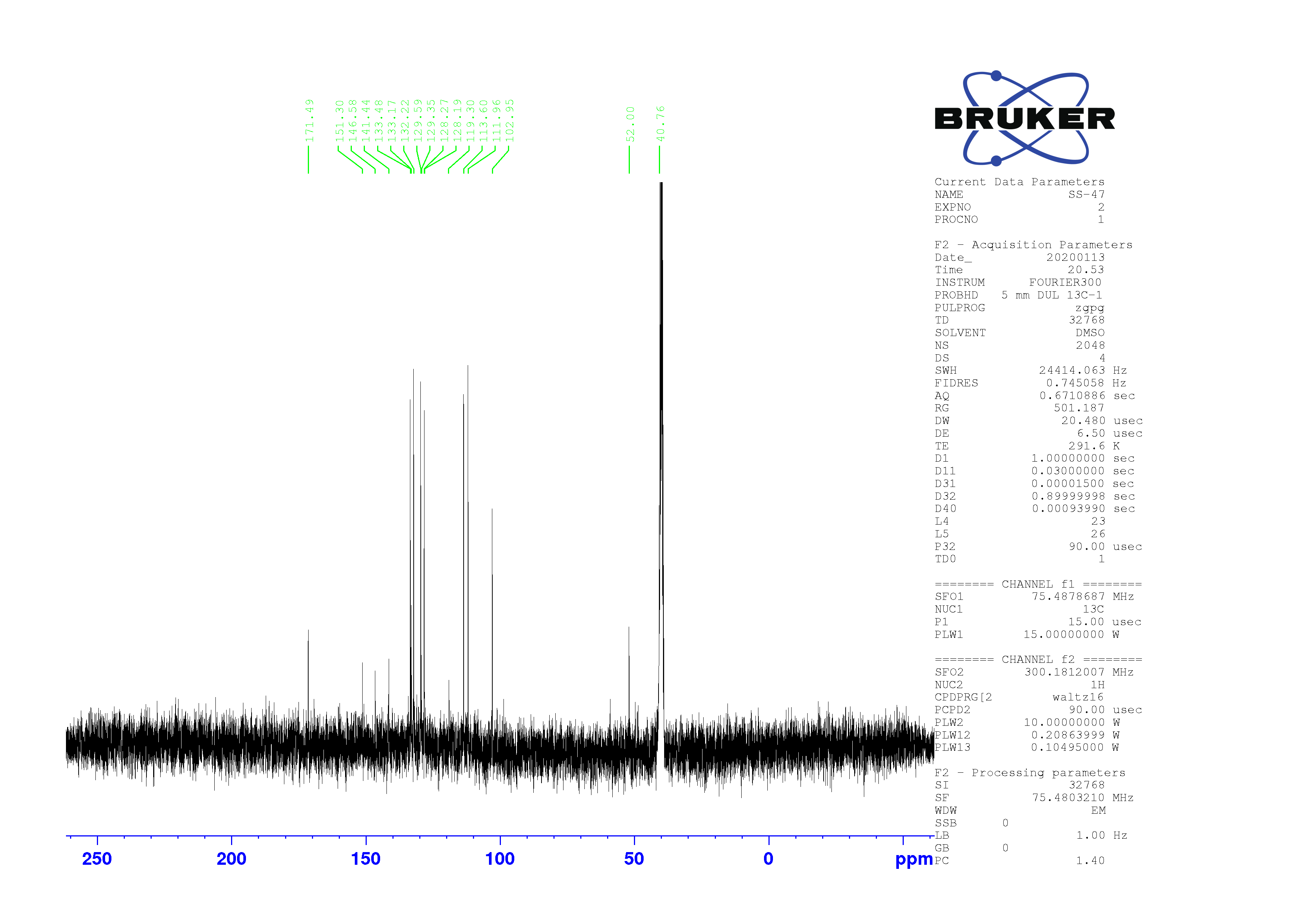


3b


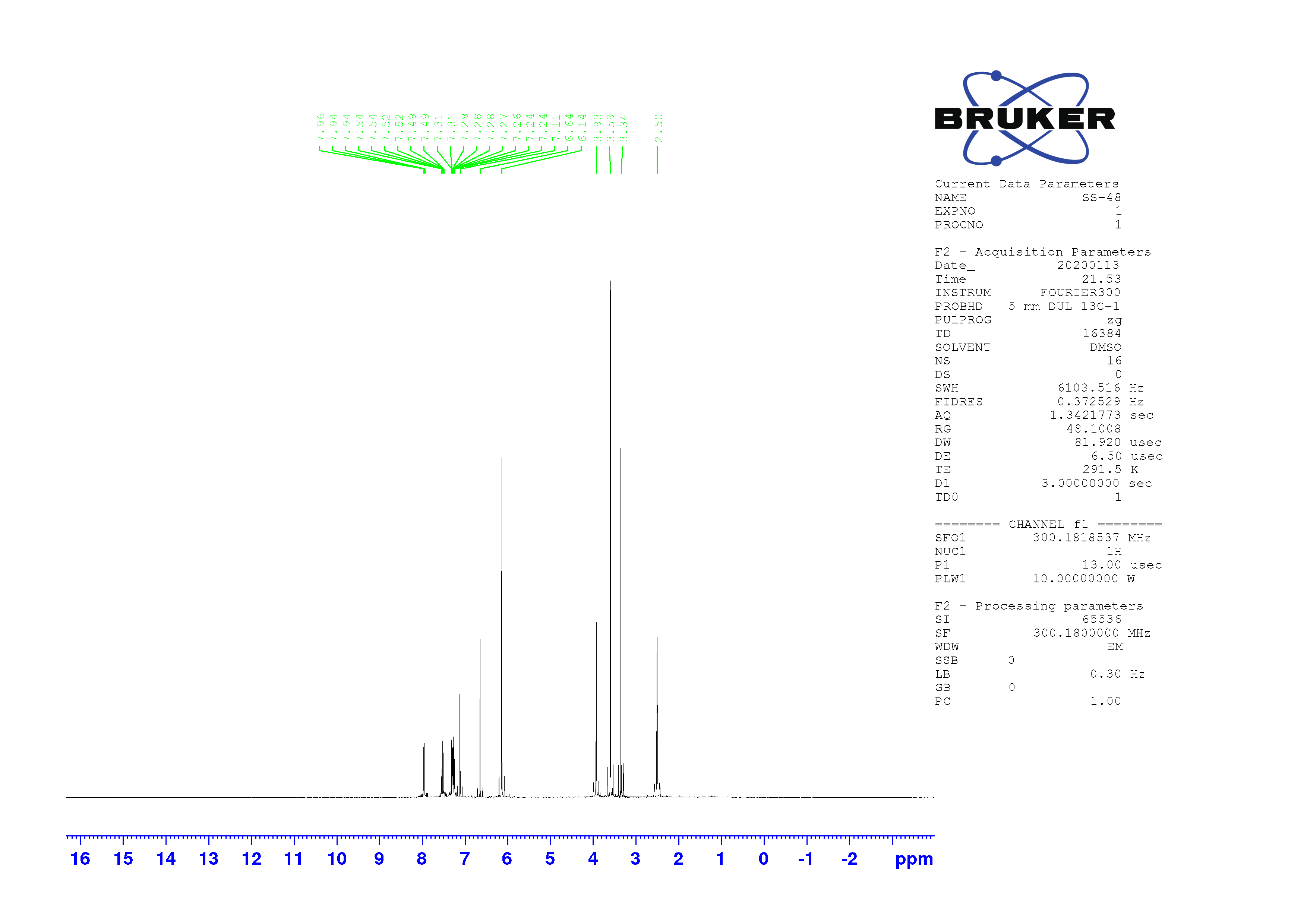


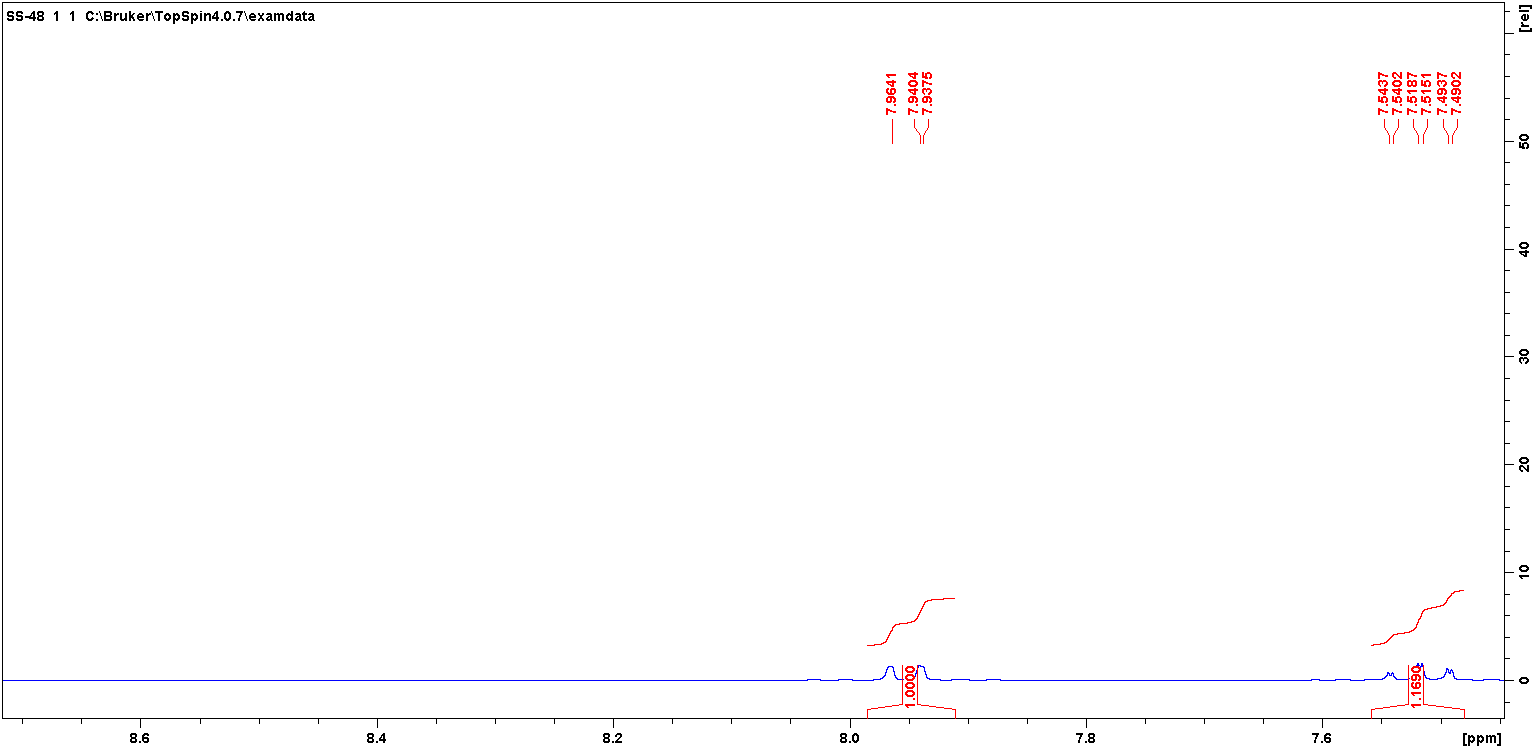


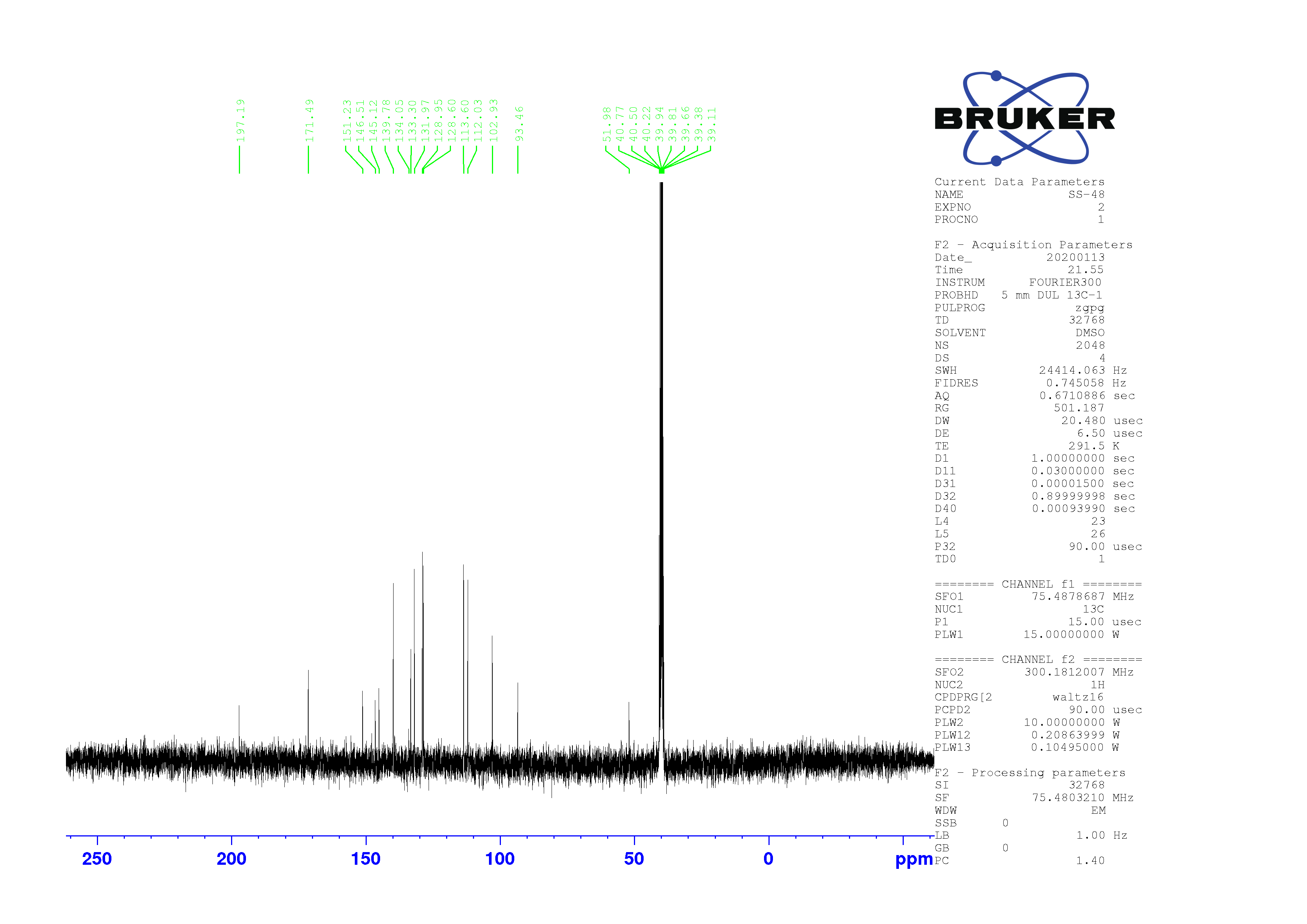


3e


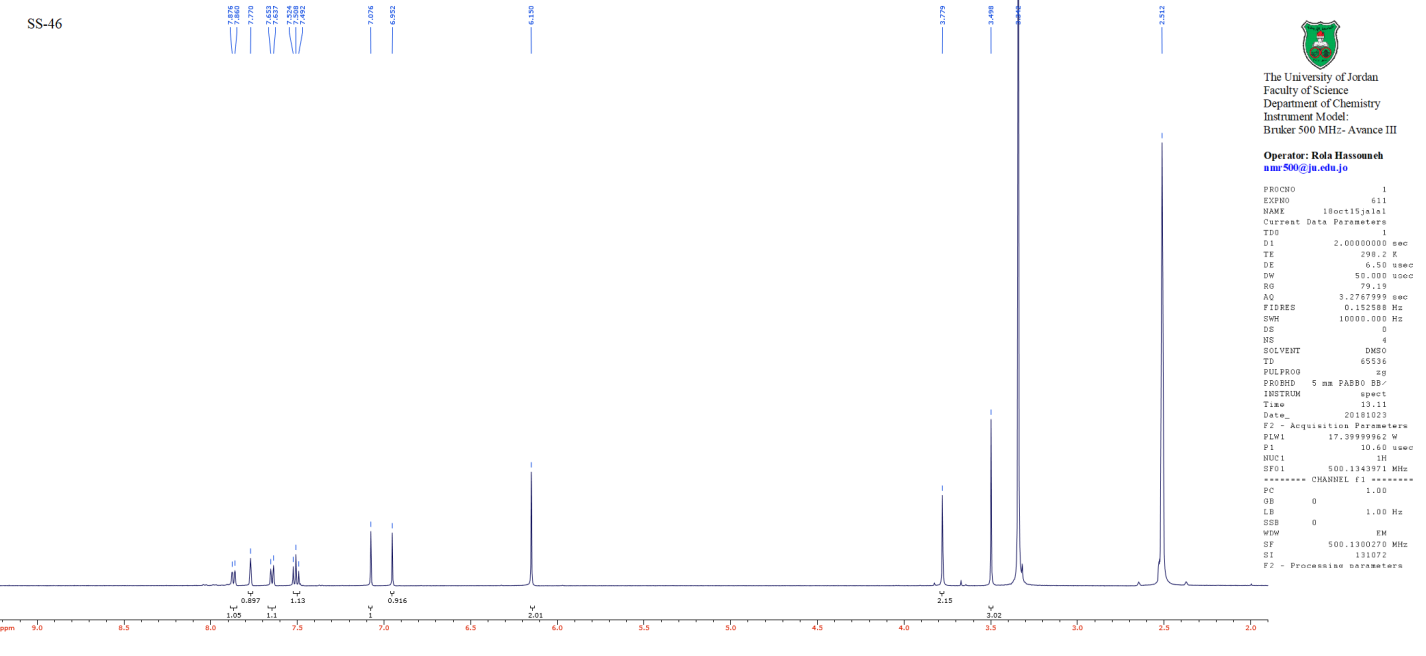


4e


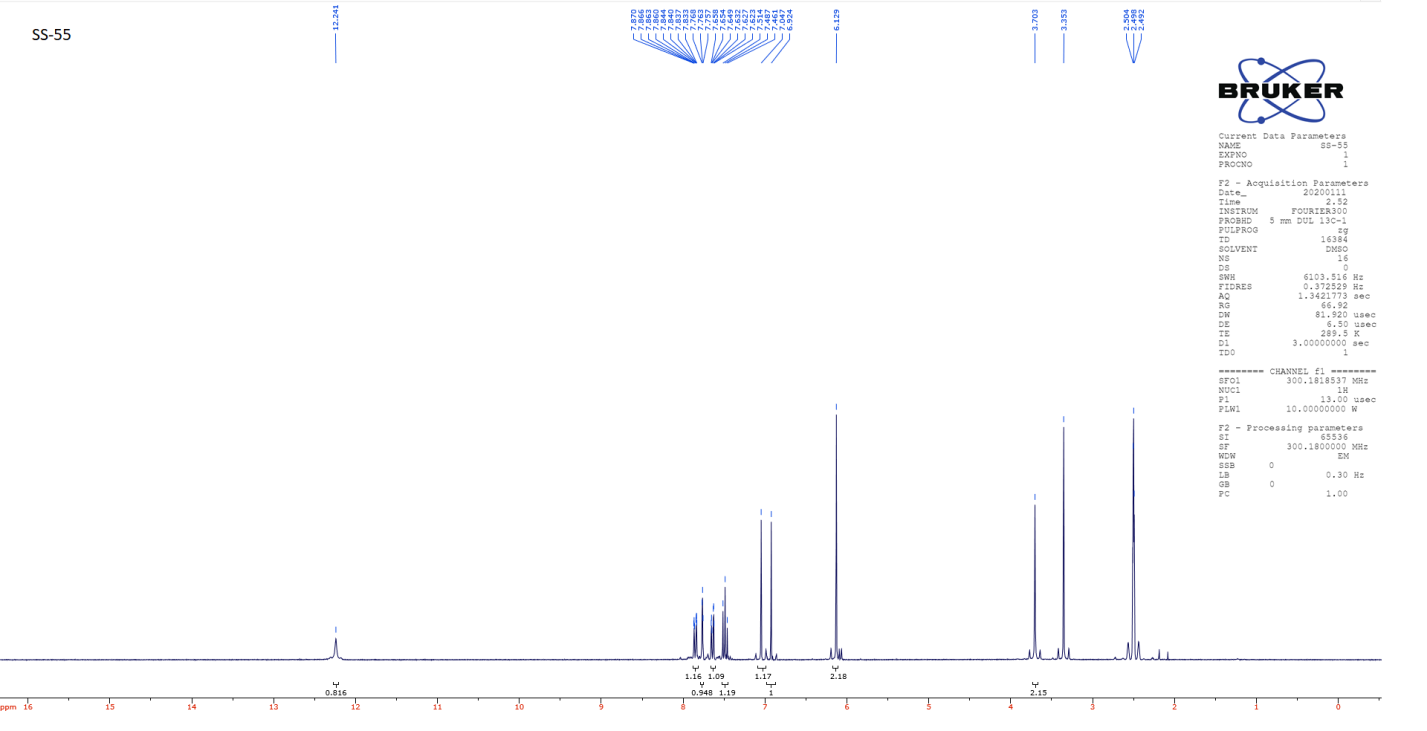


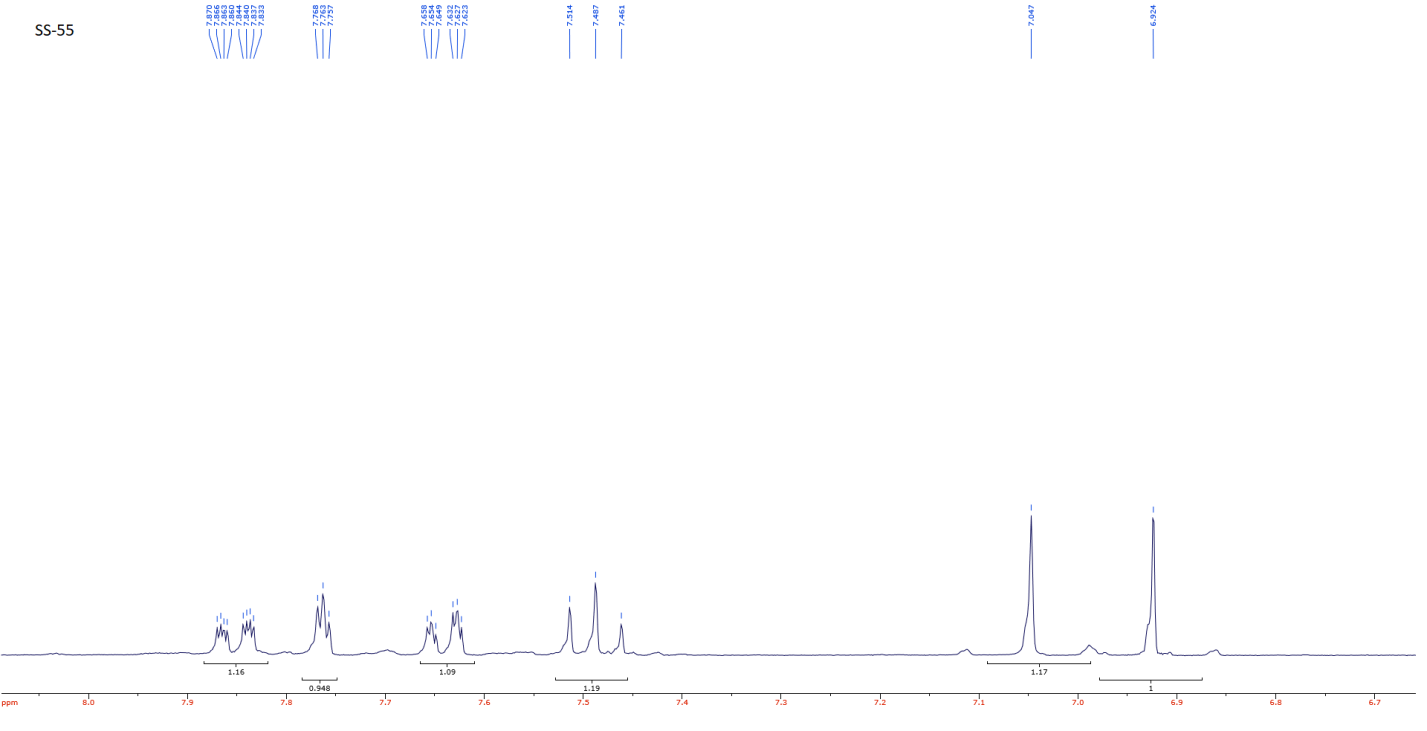


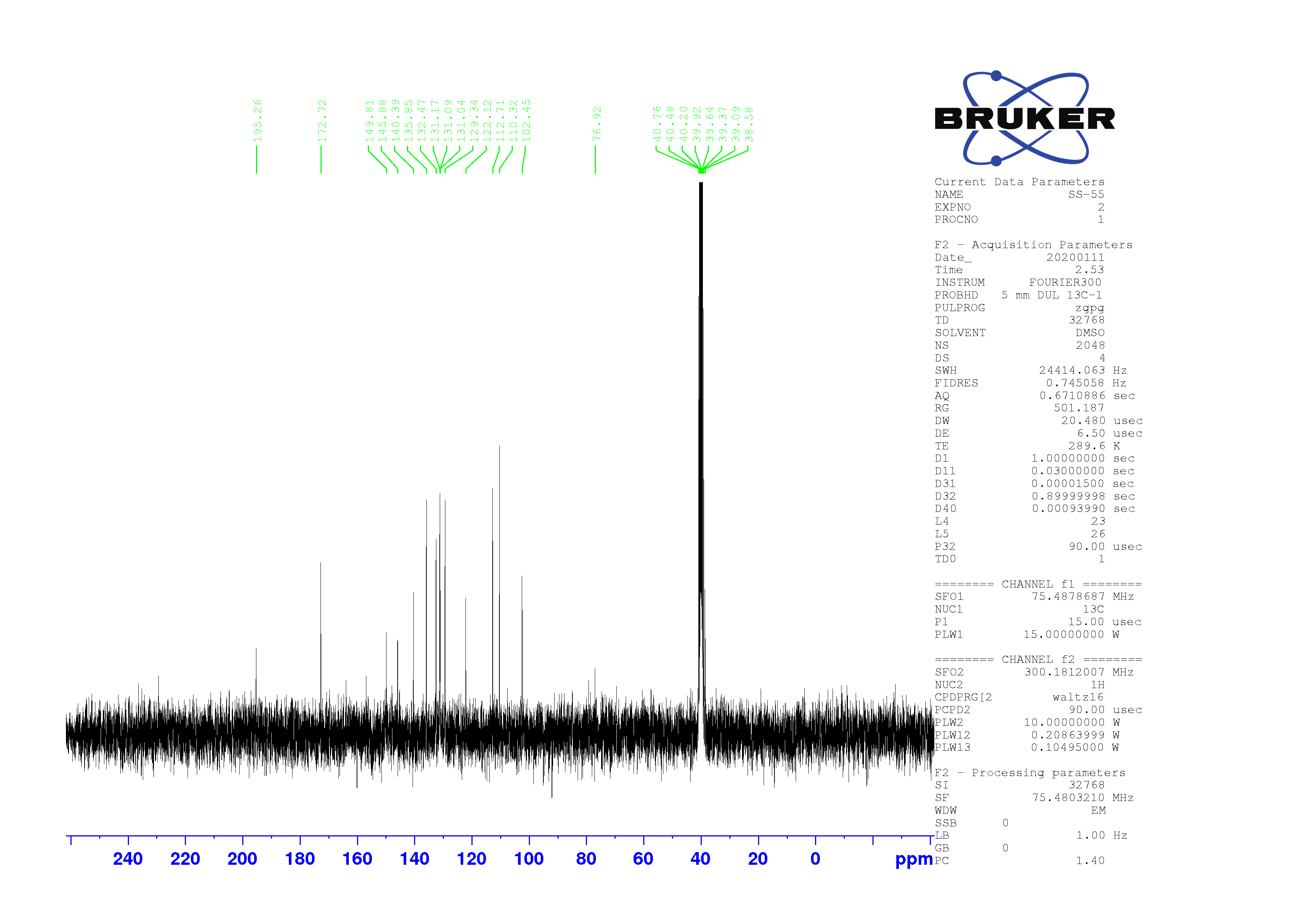


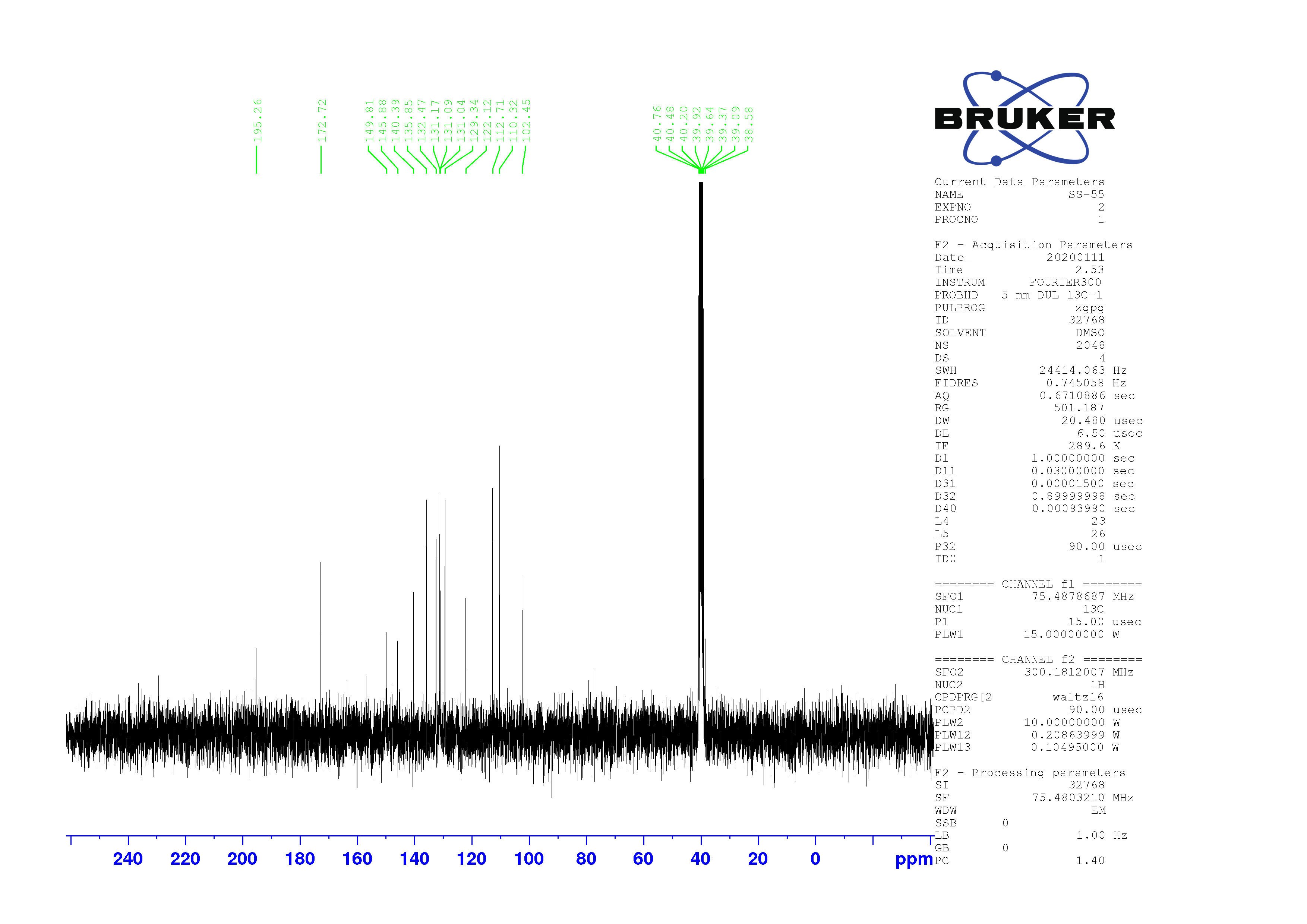


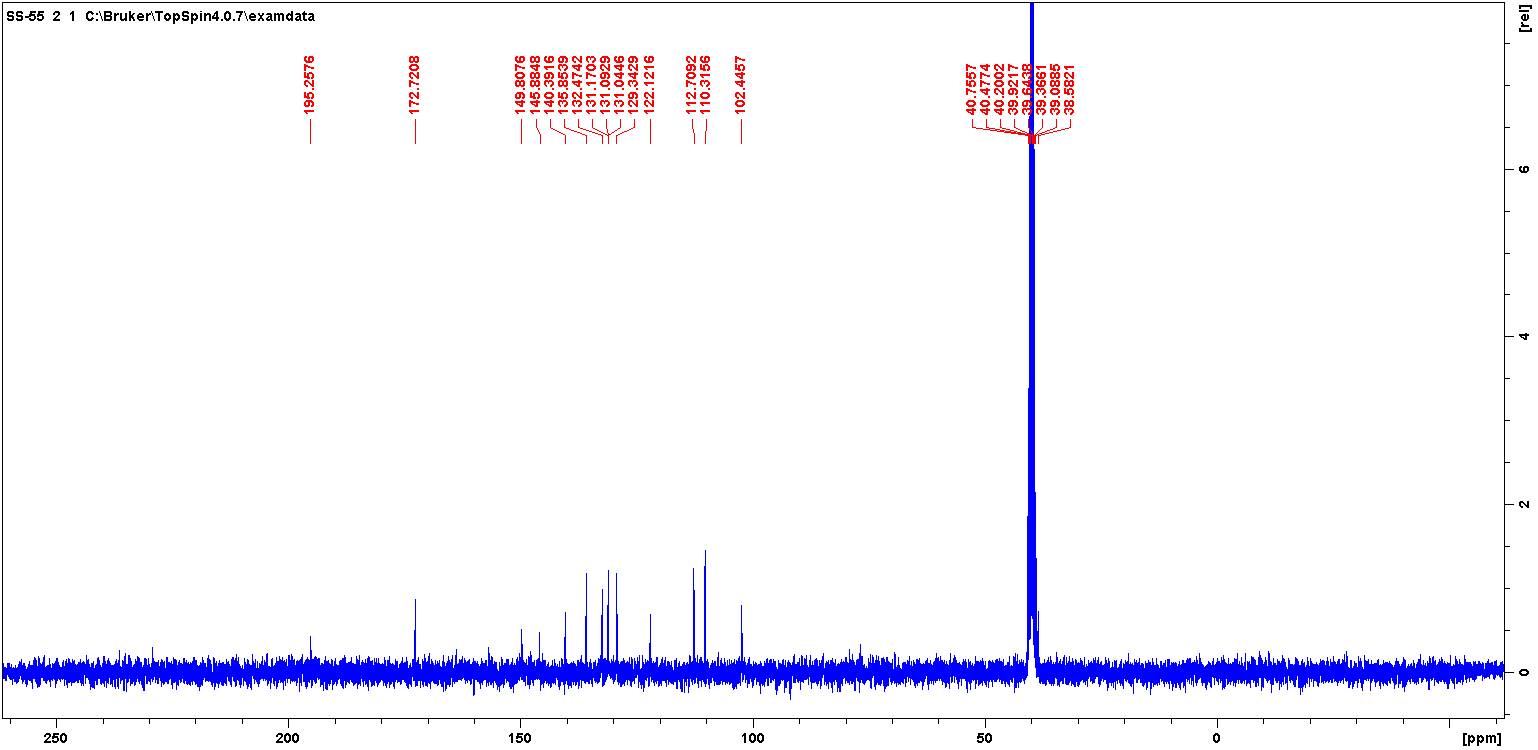


4d


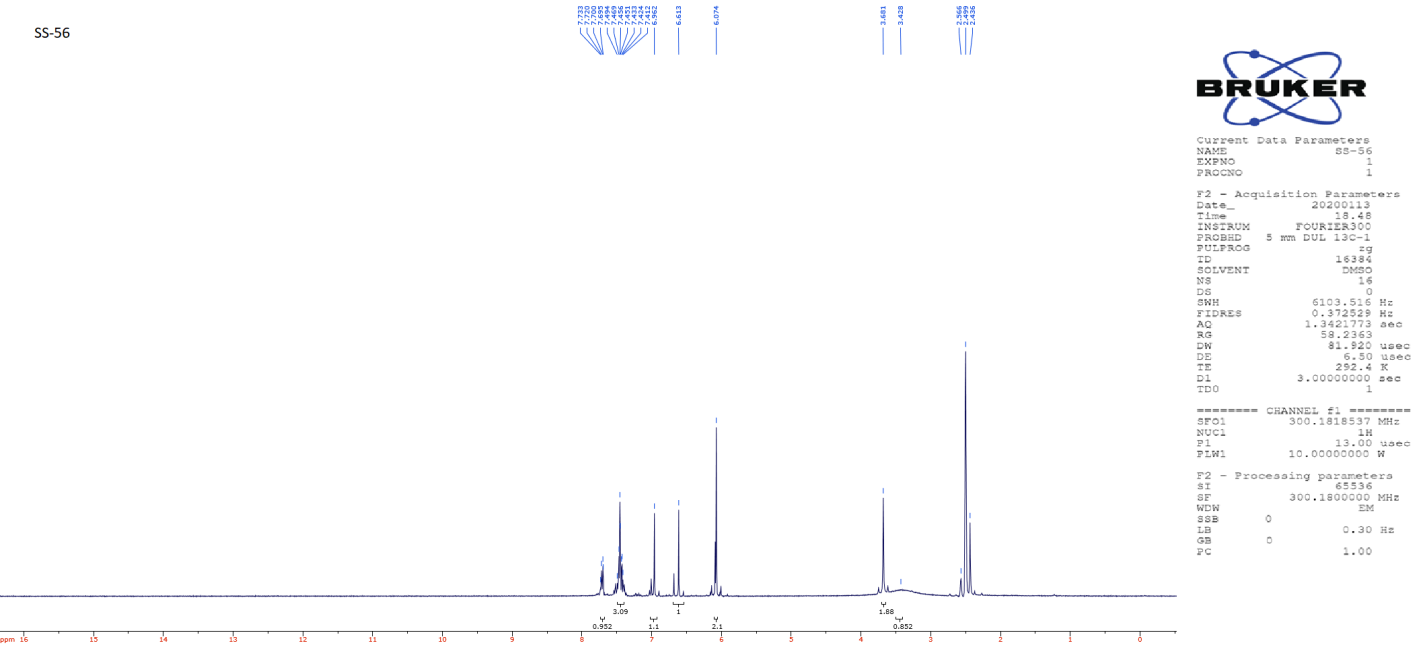


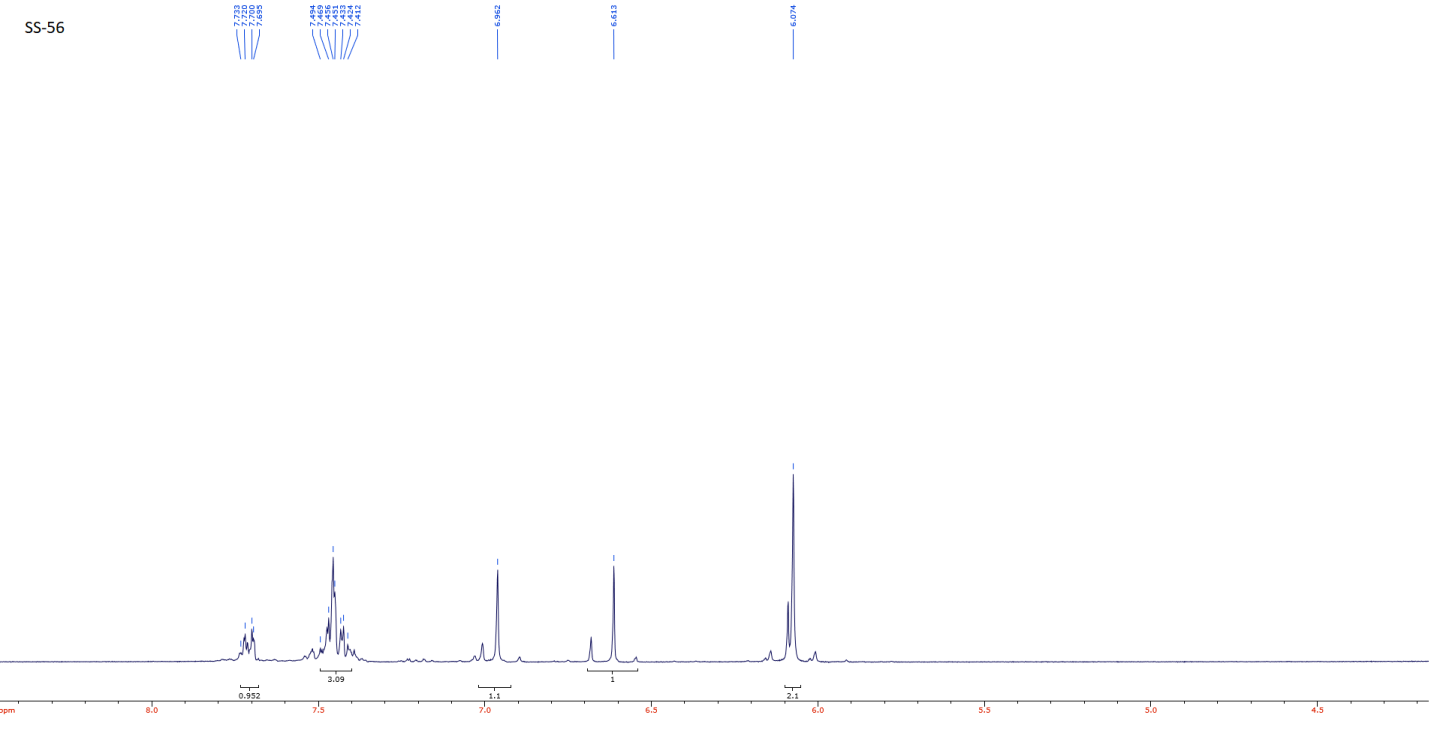


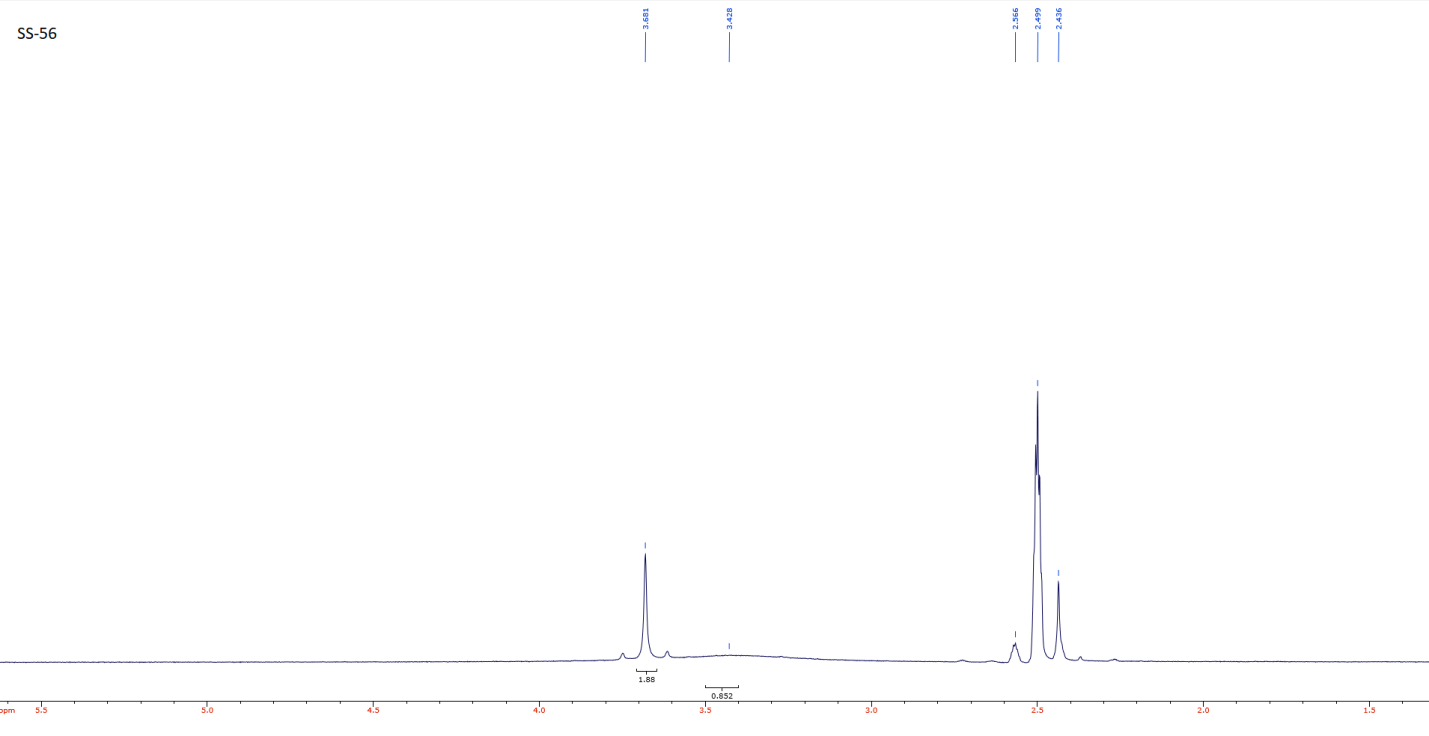


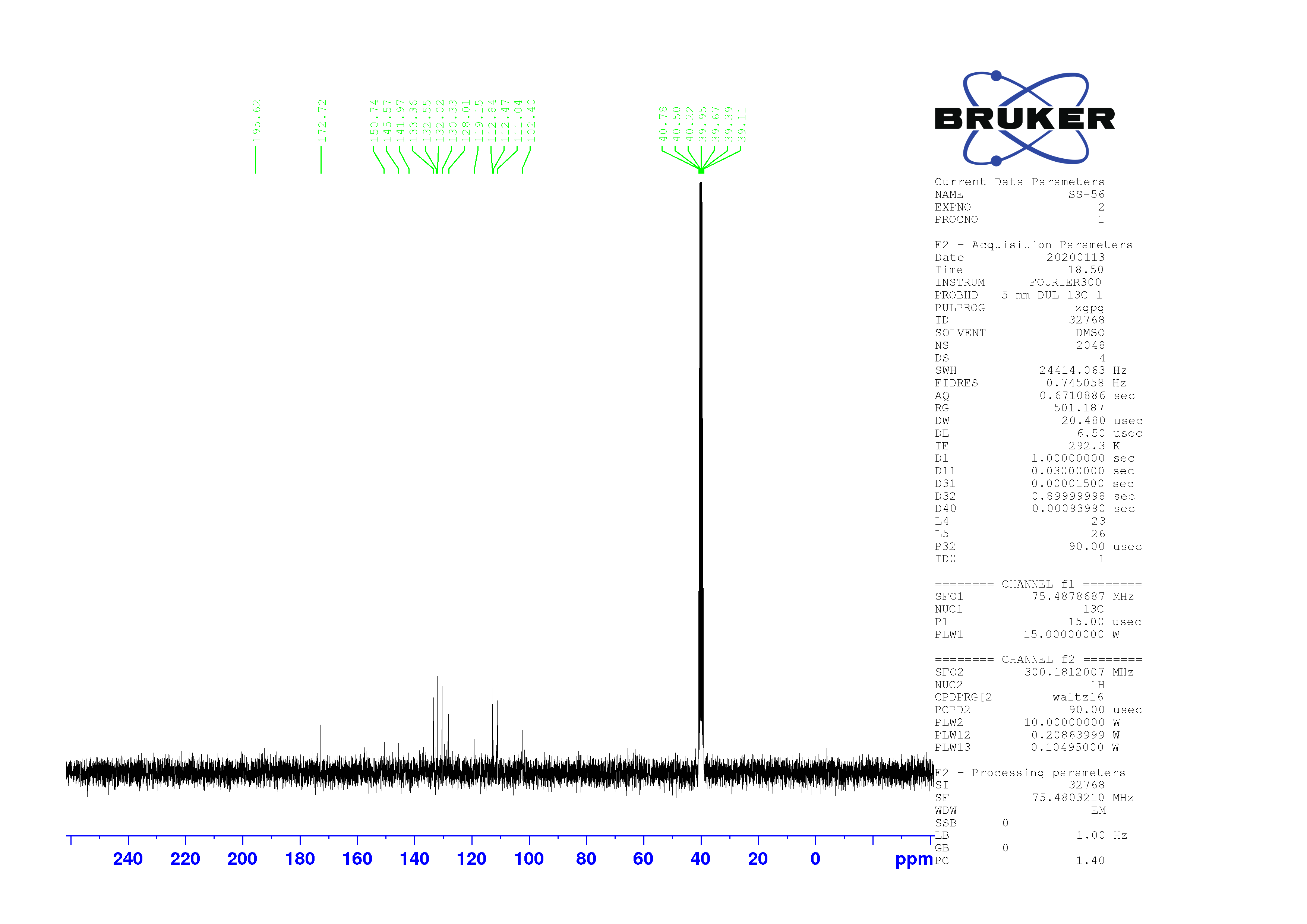


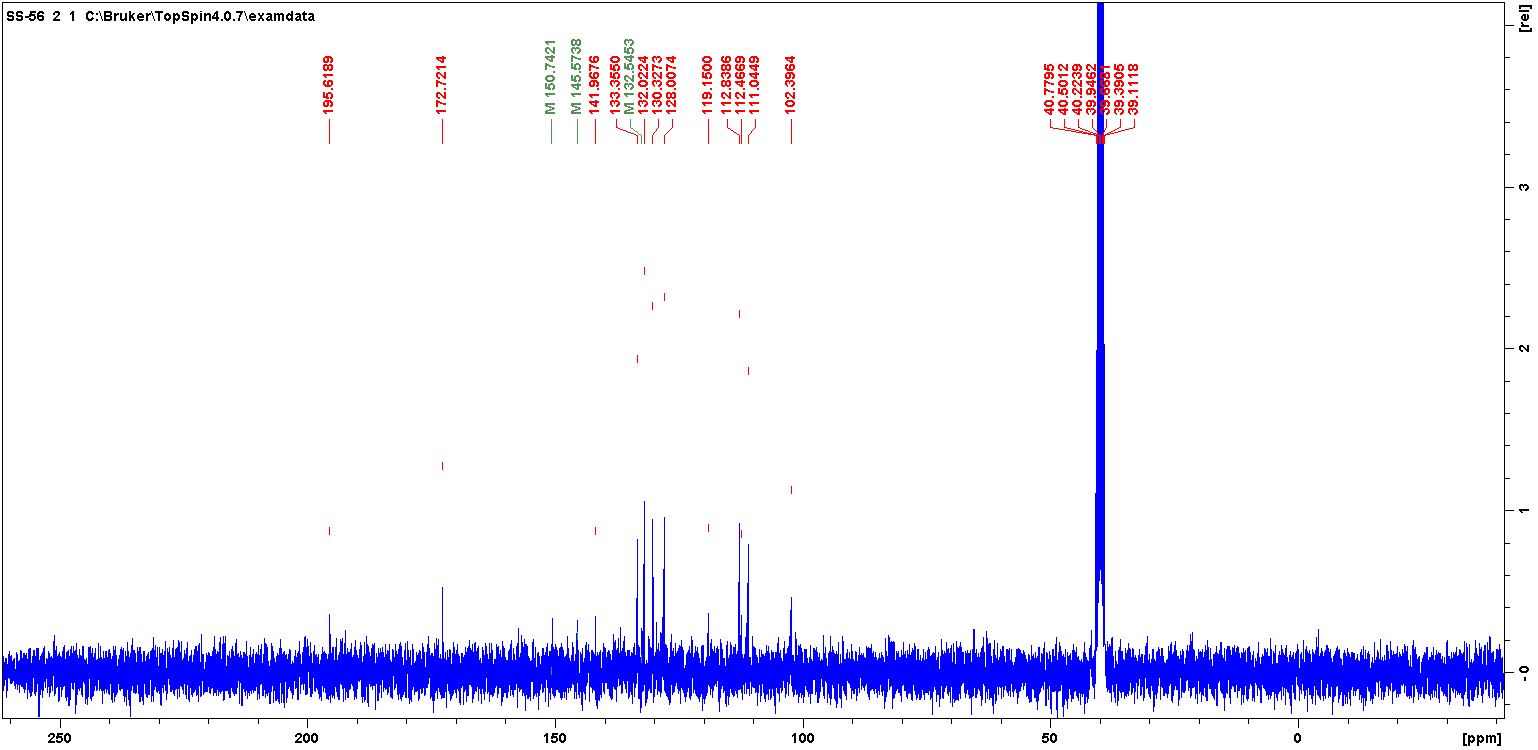


4b


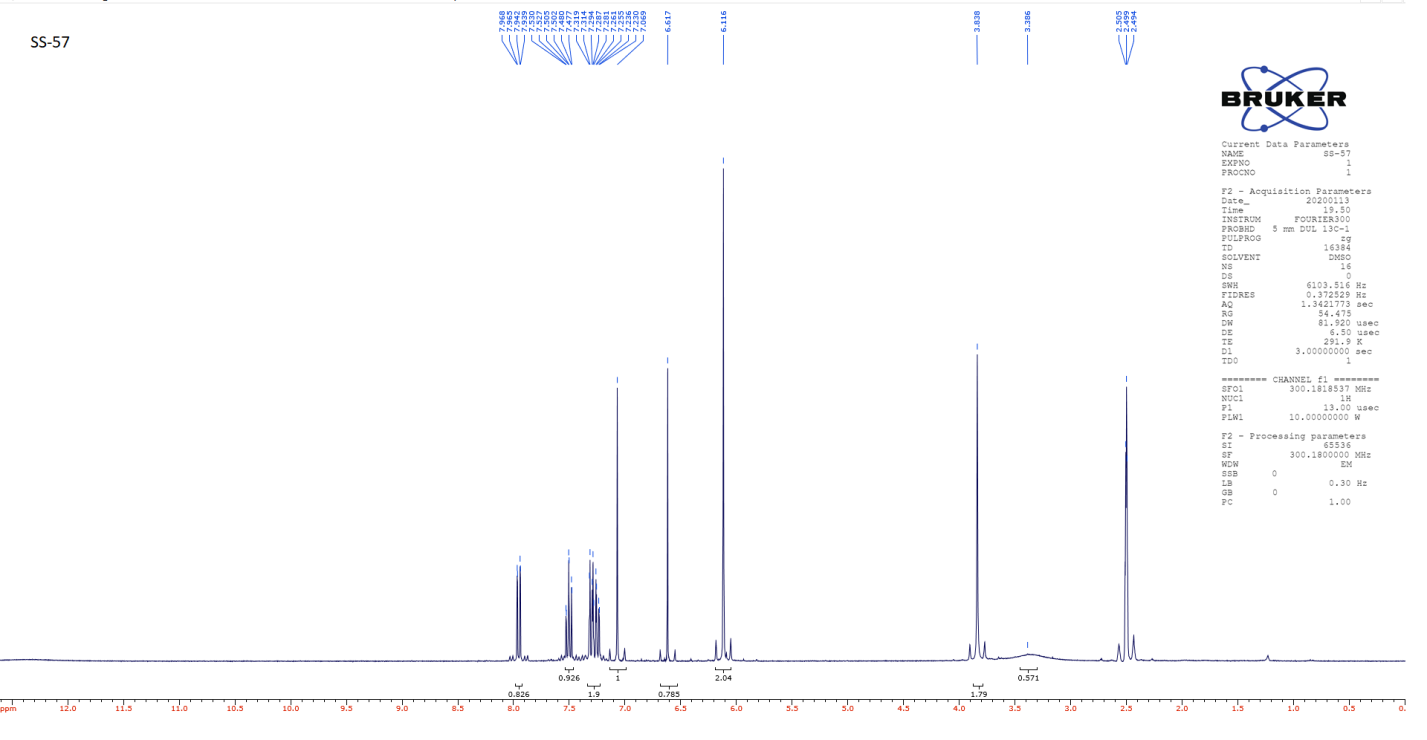


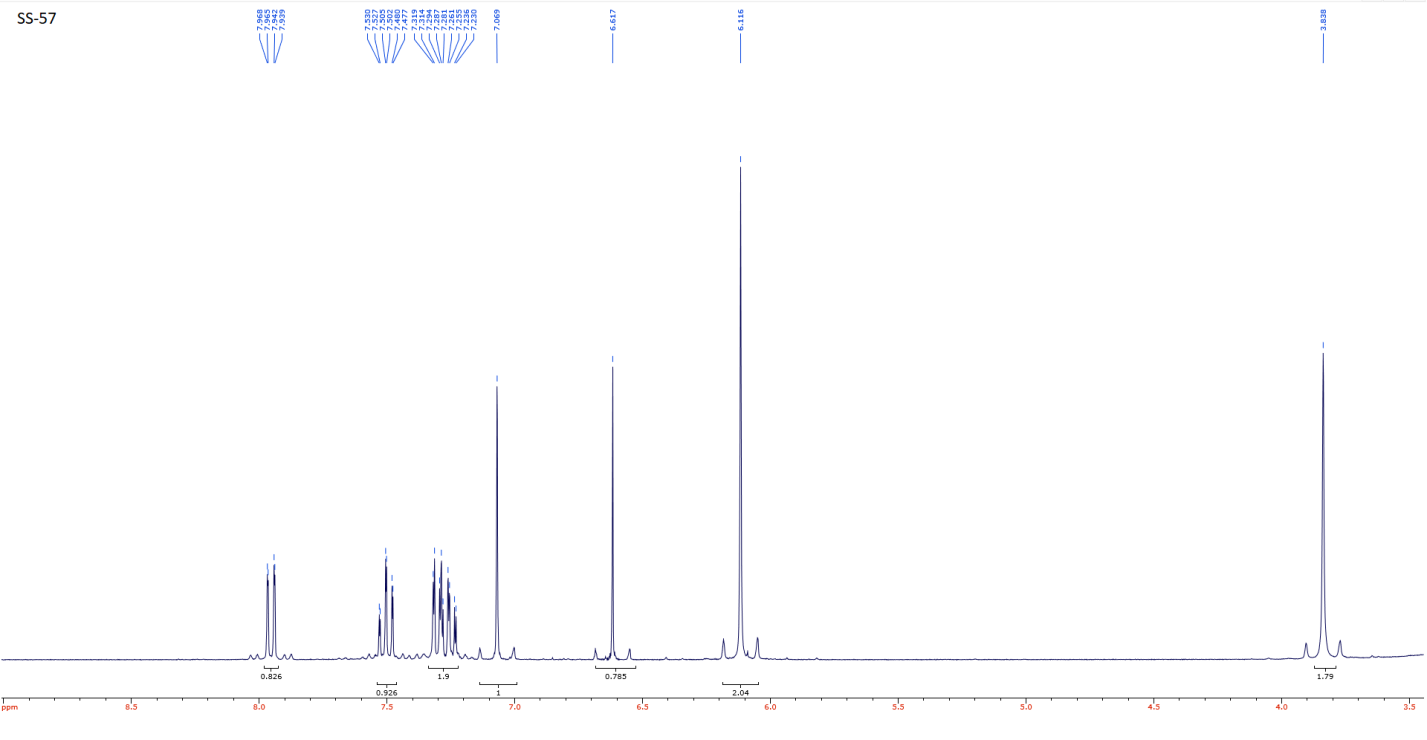


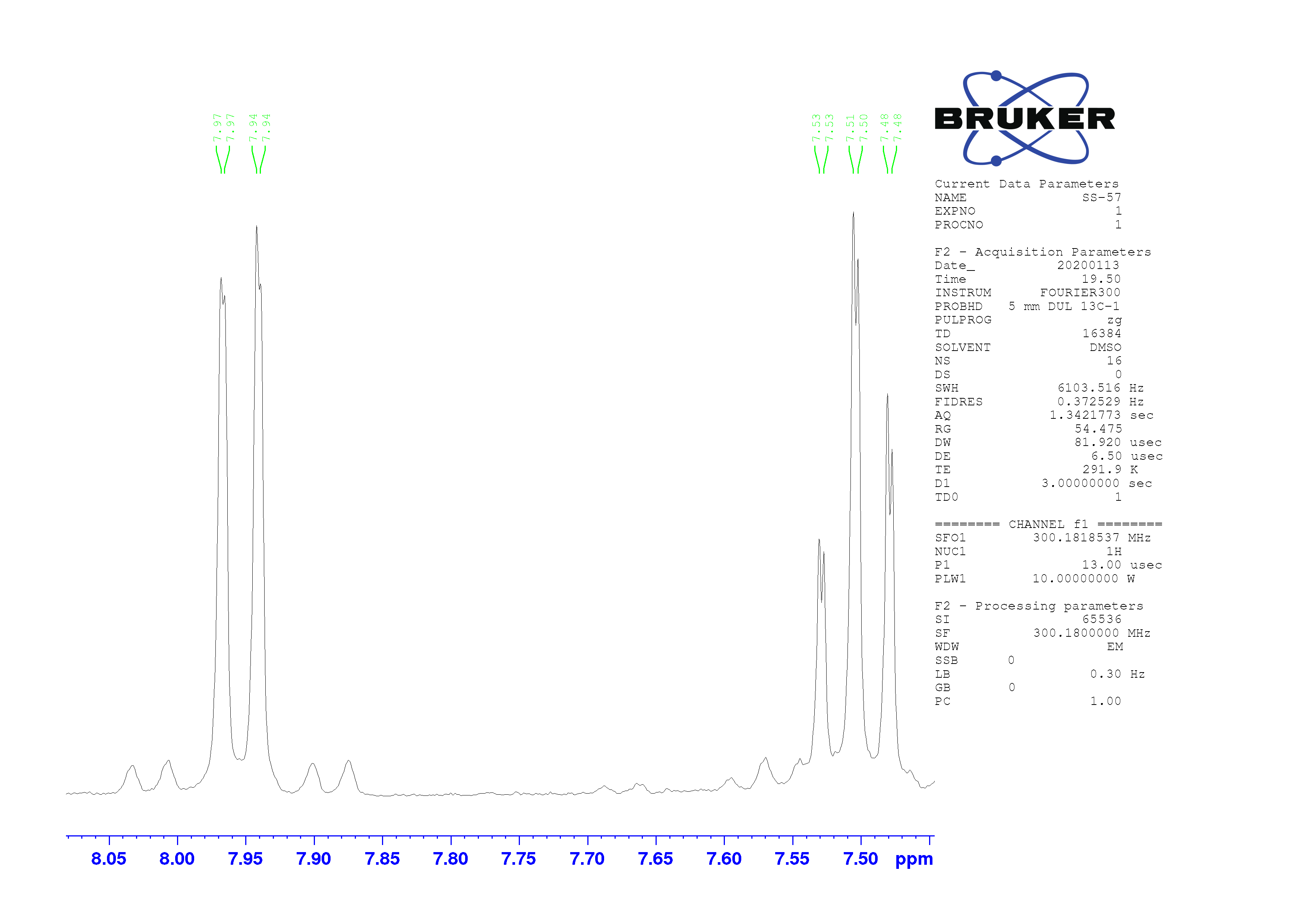


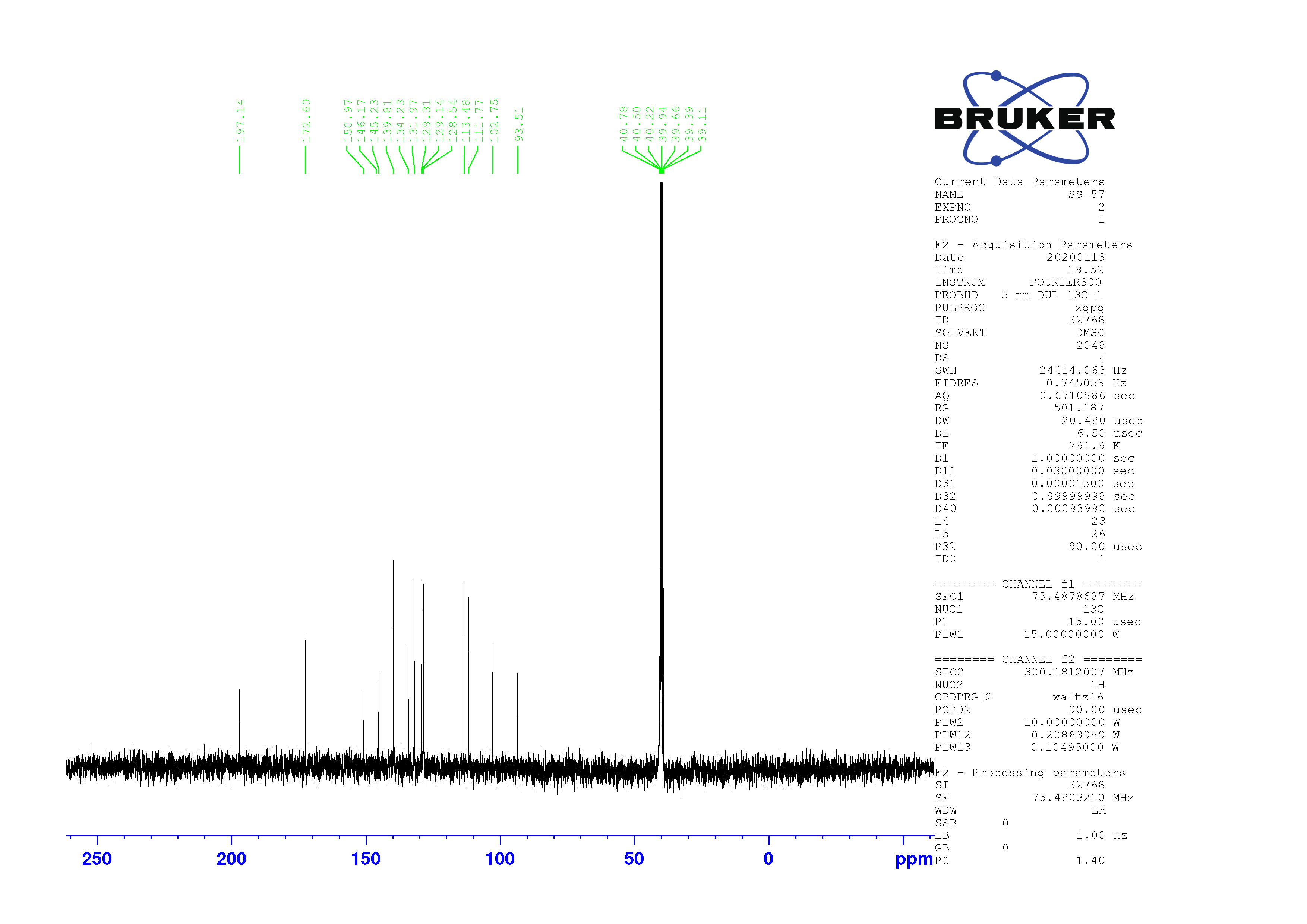


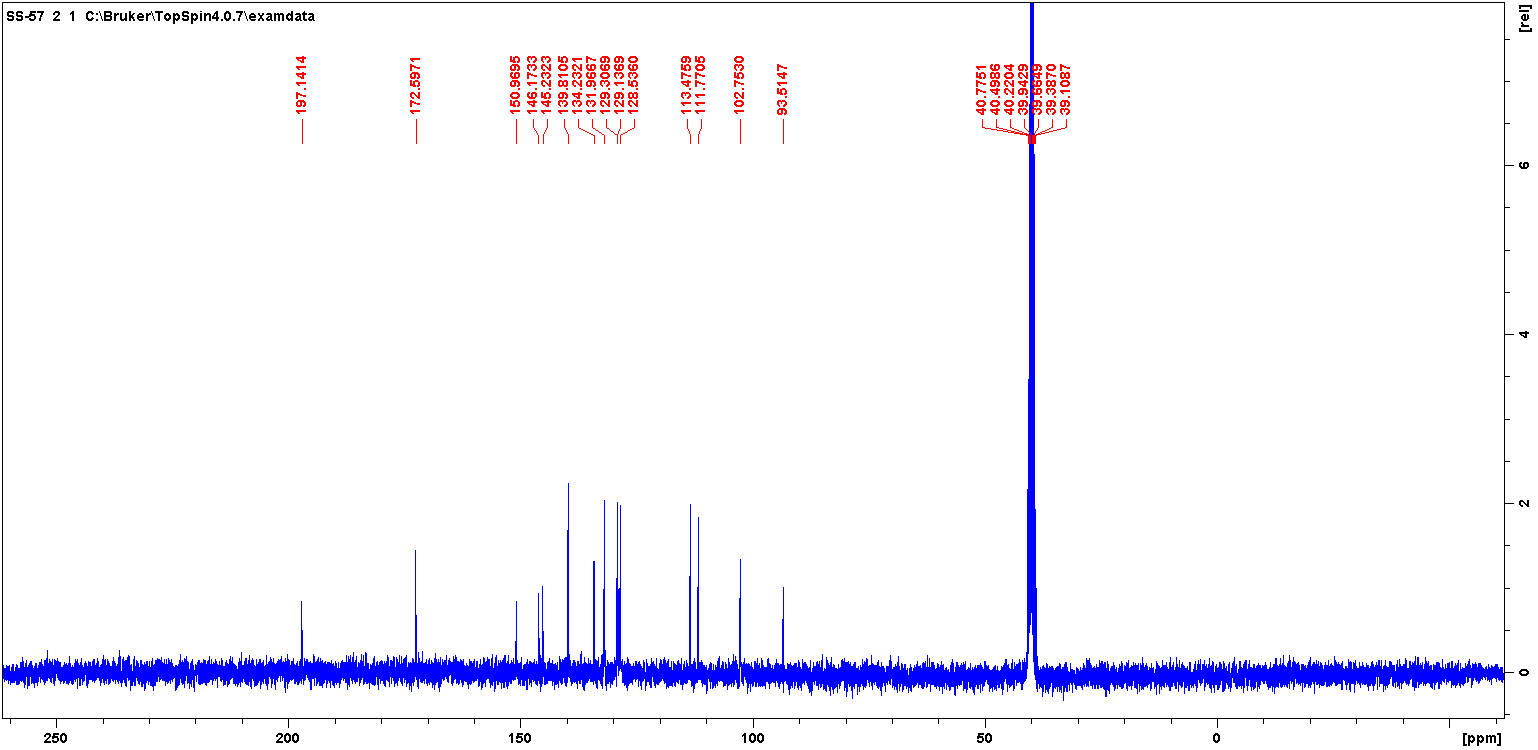


4c


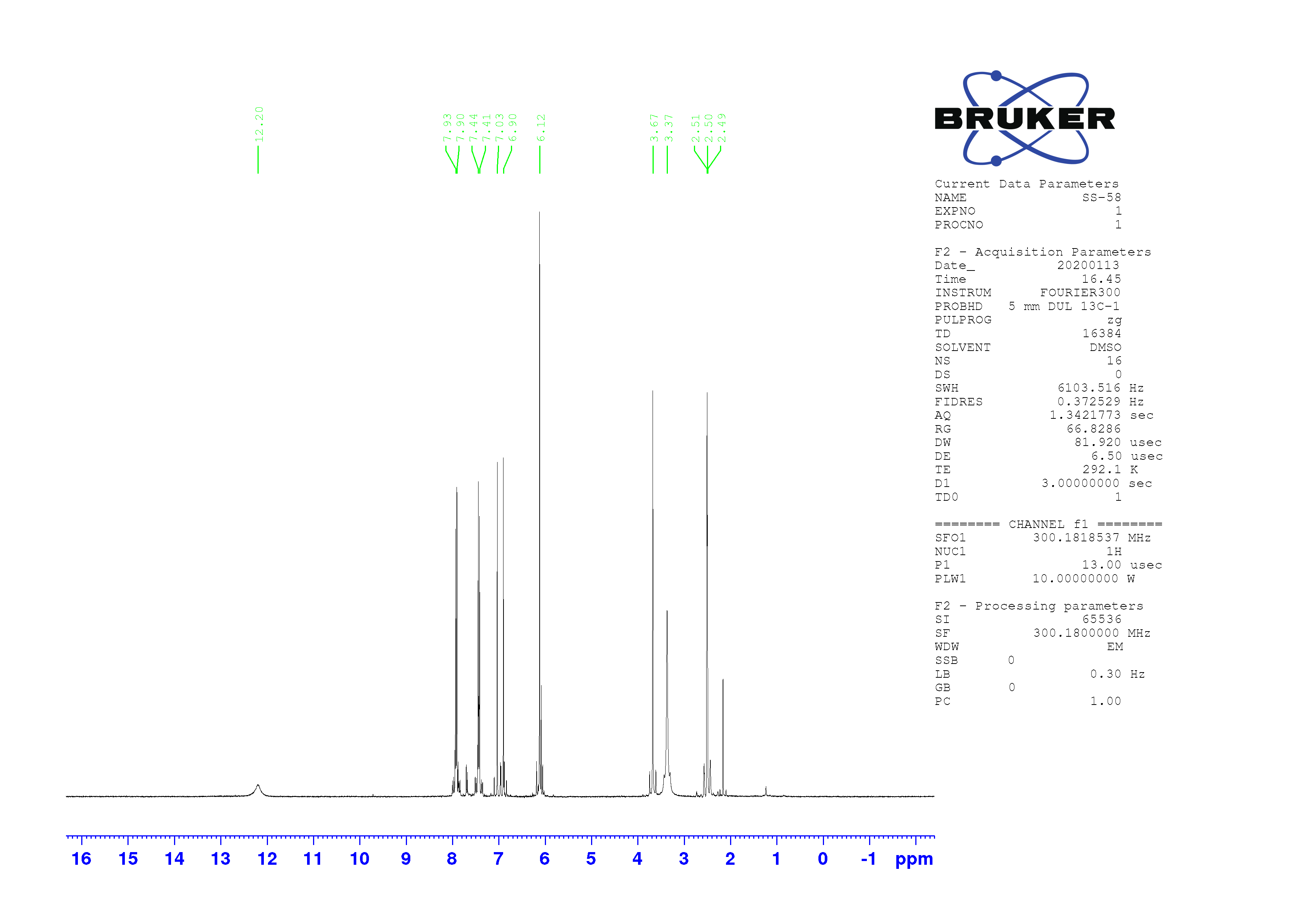


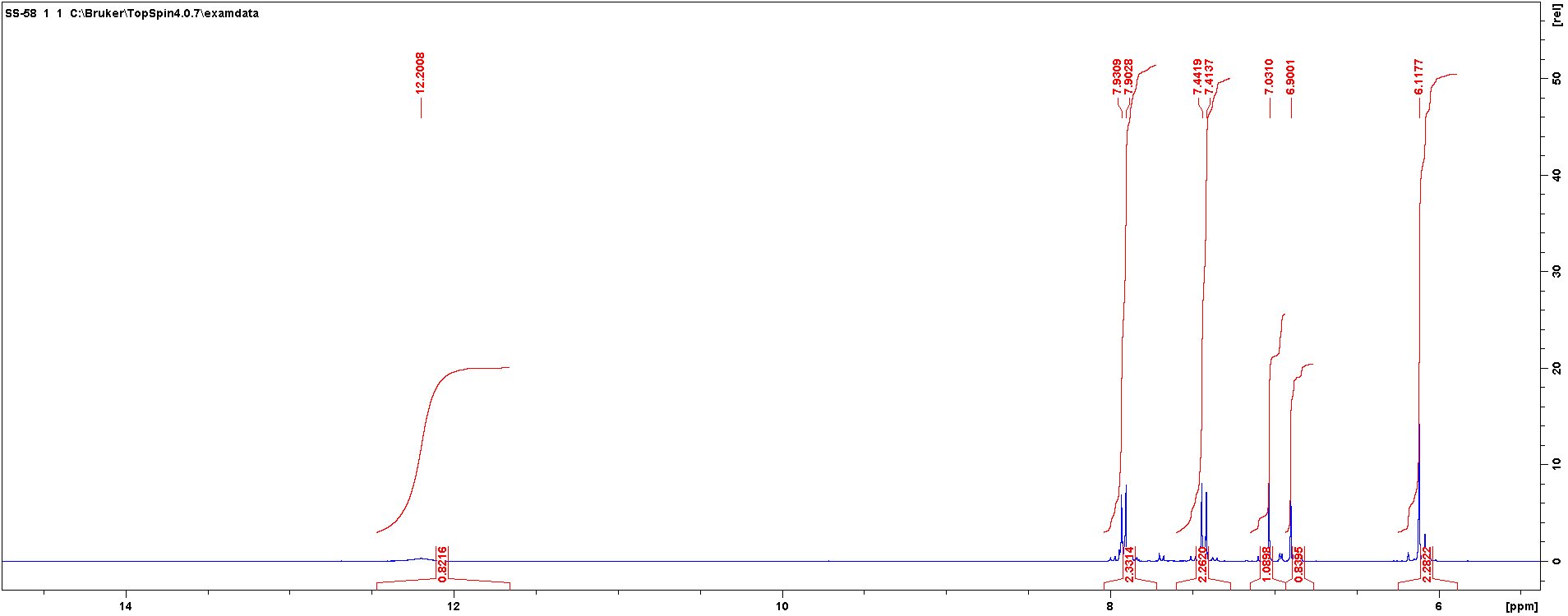


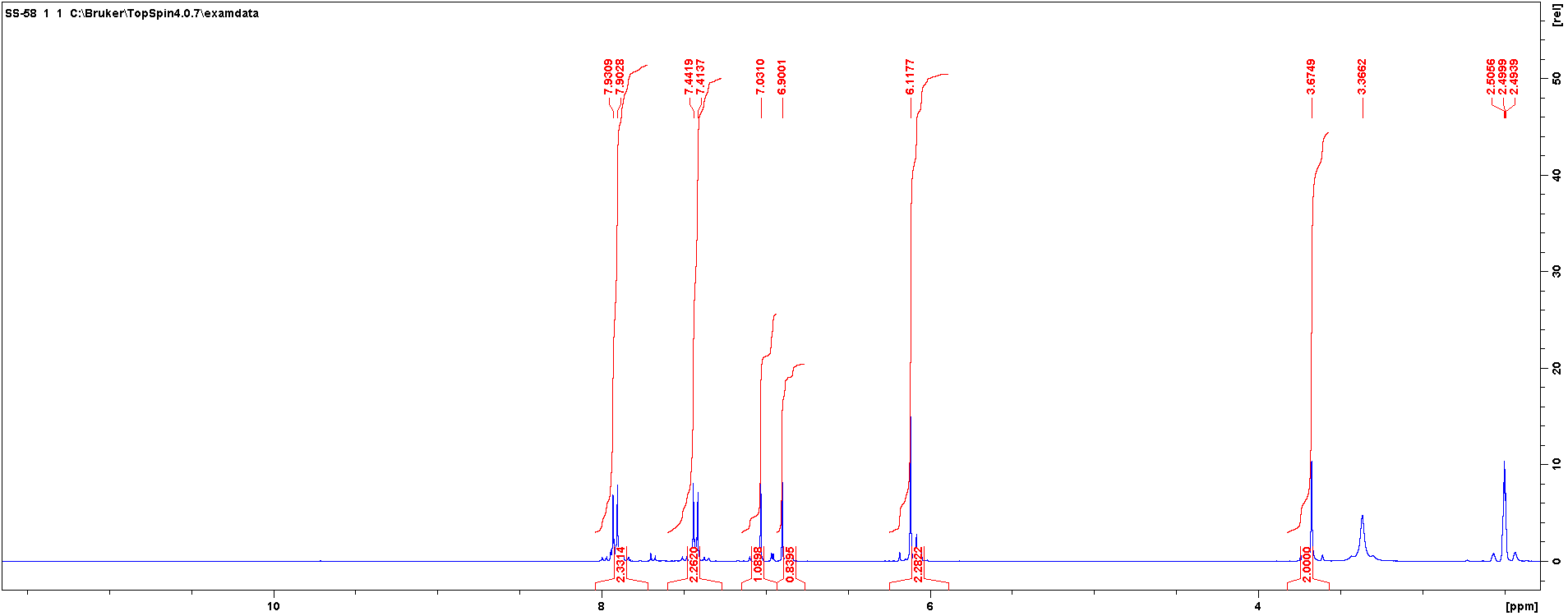


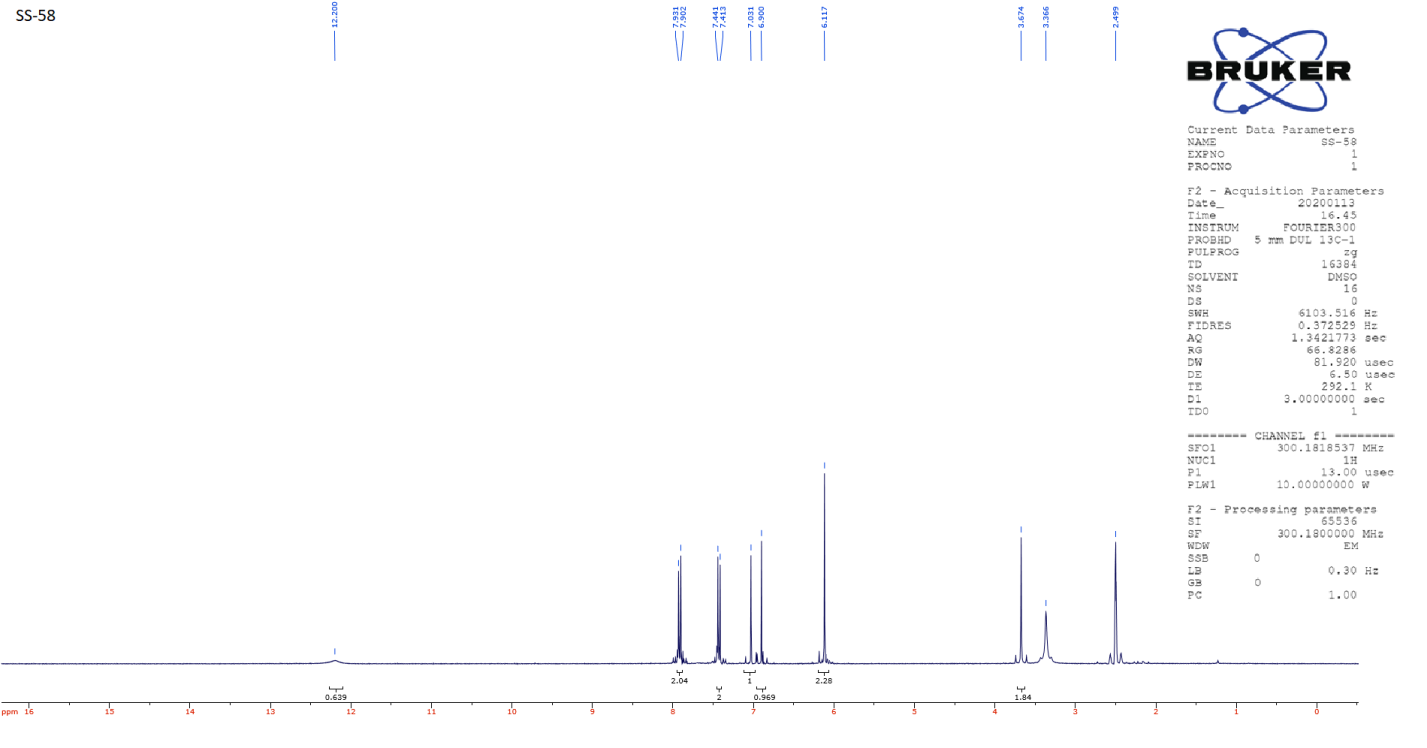


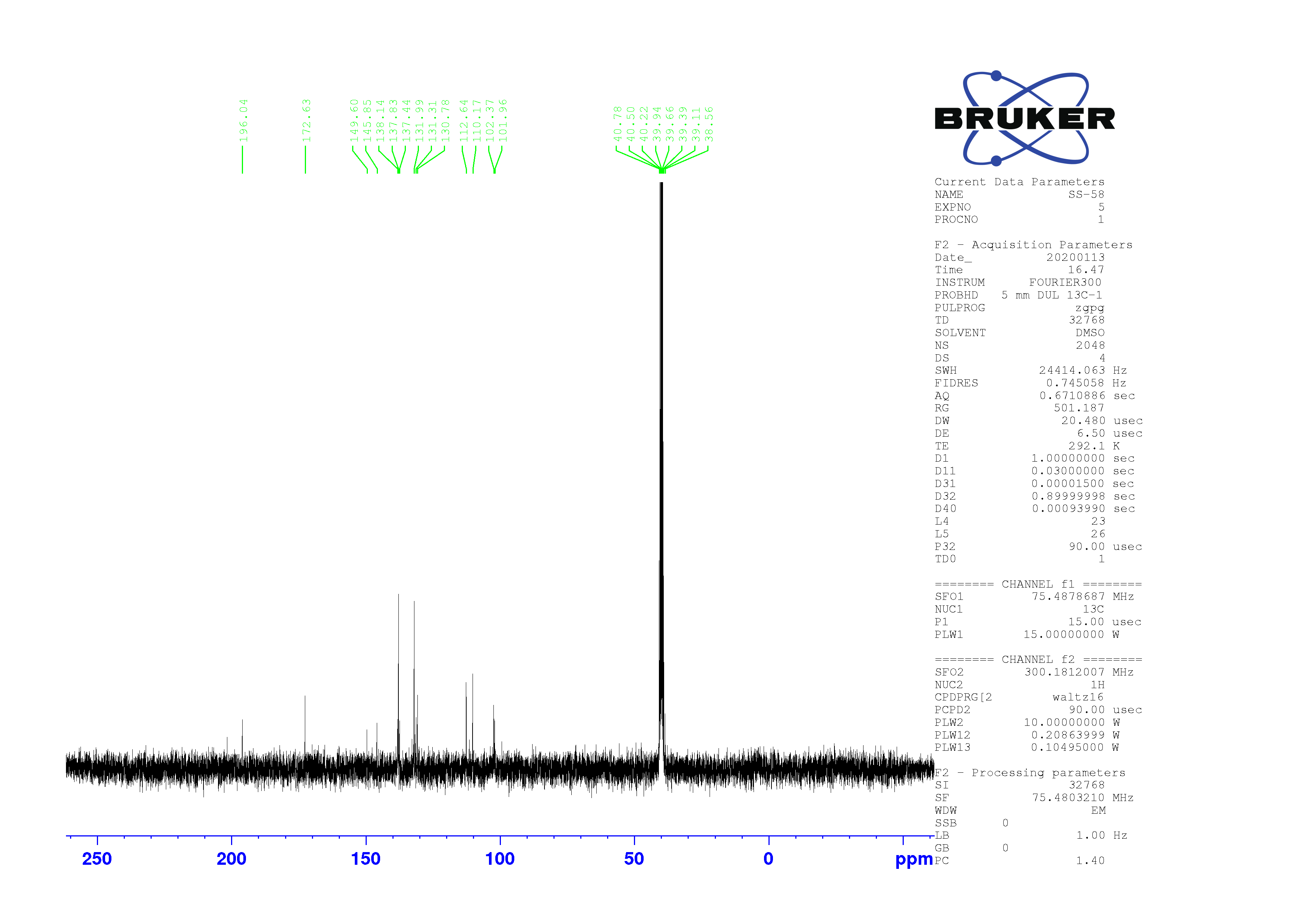


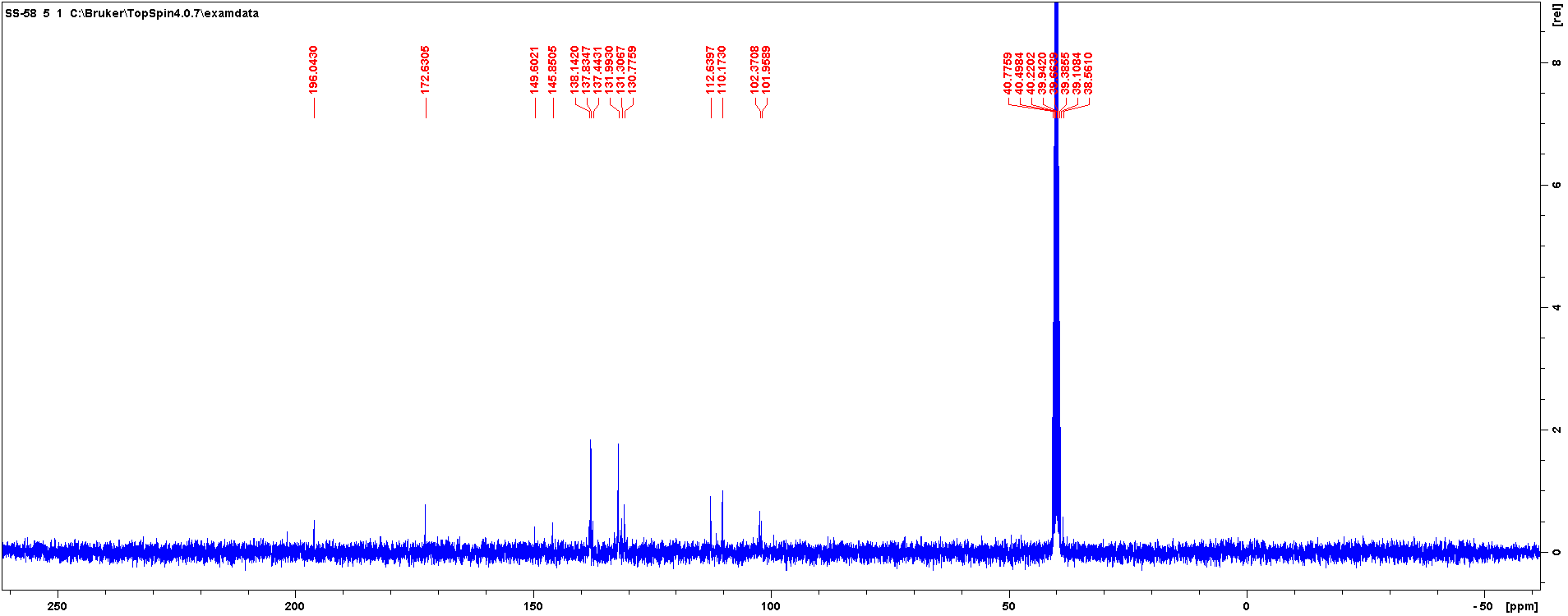


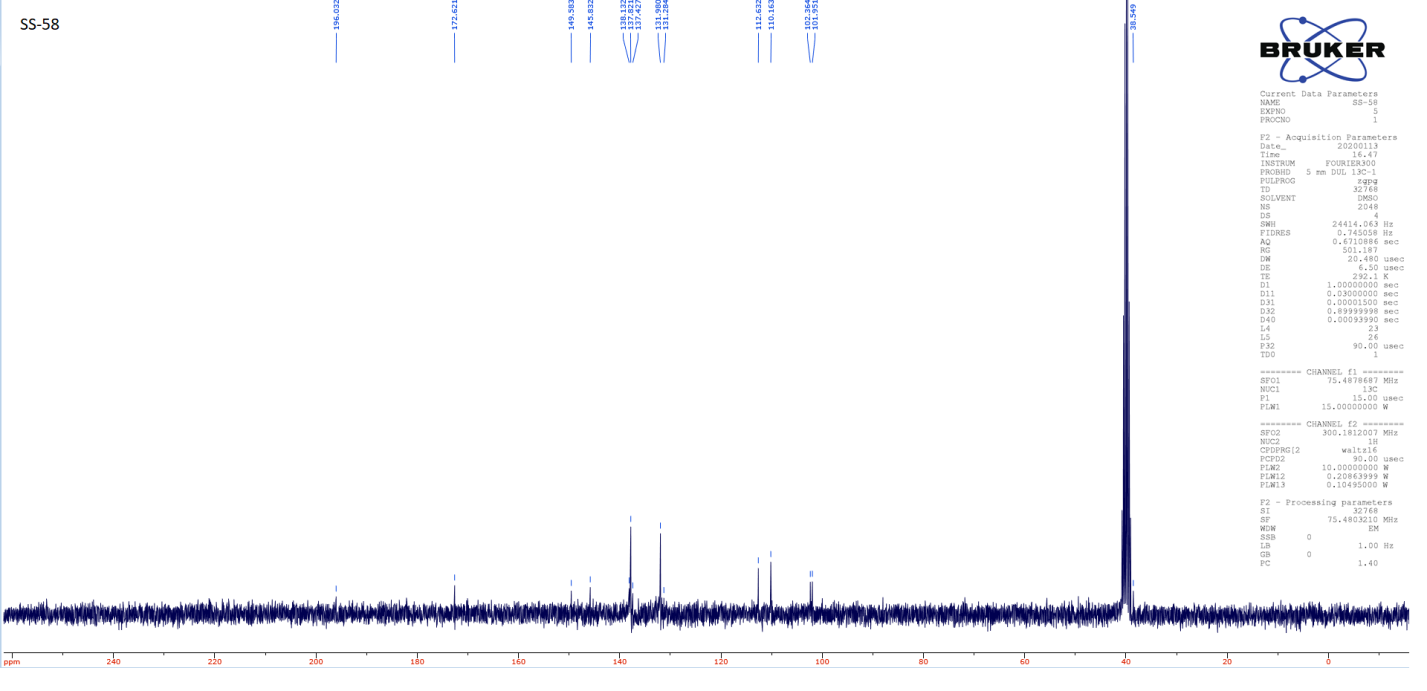


4f


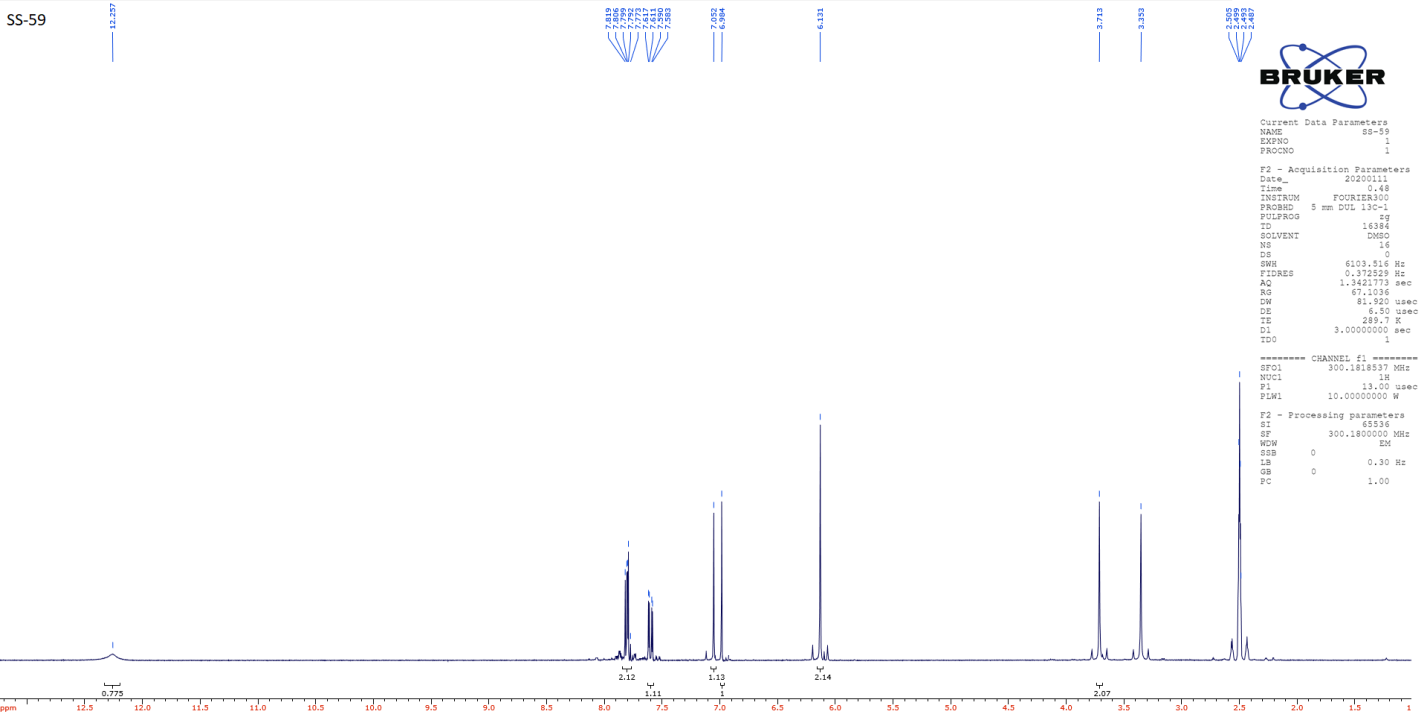


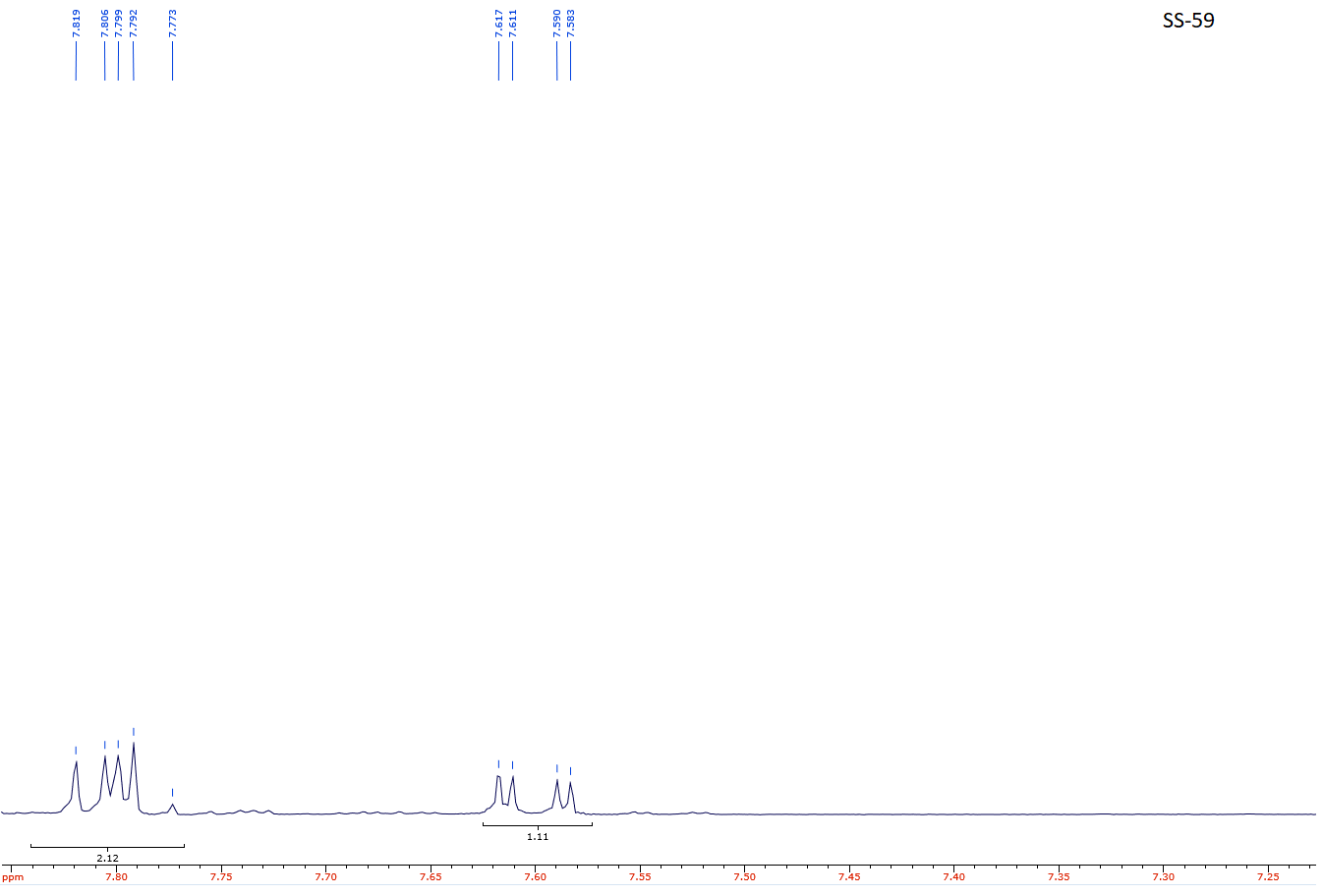


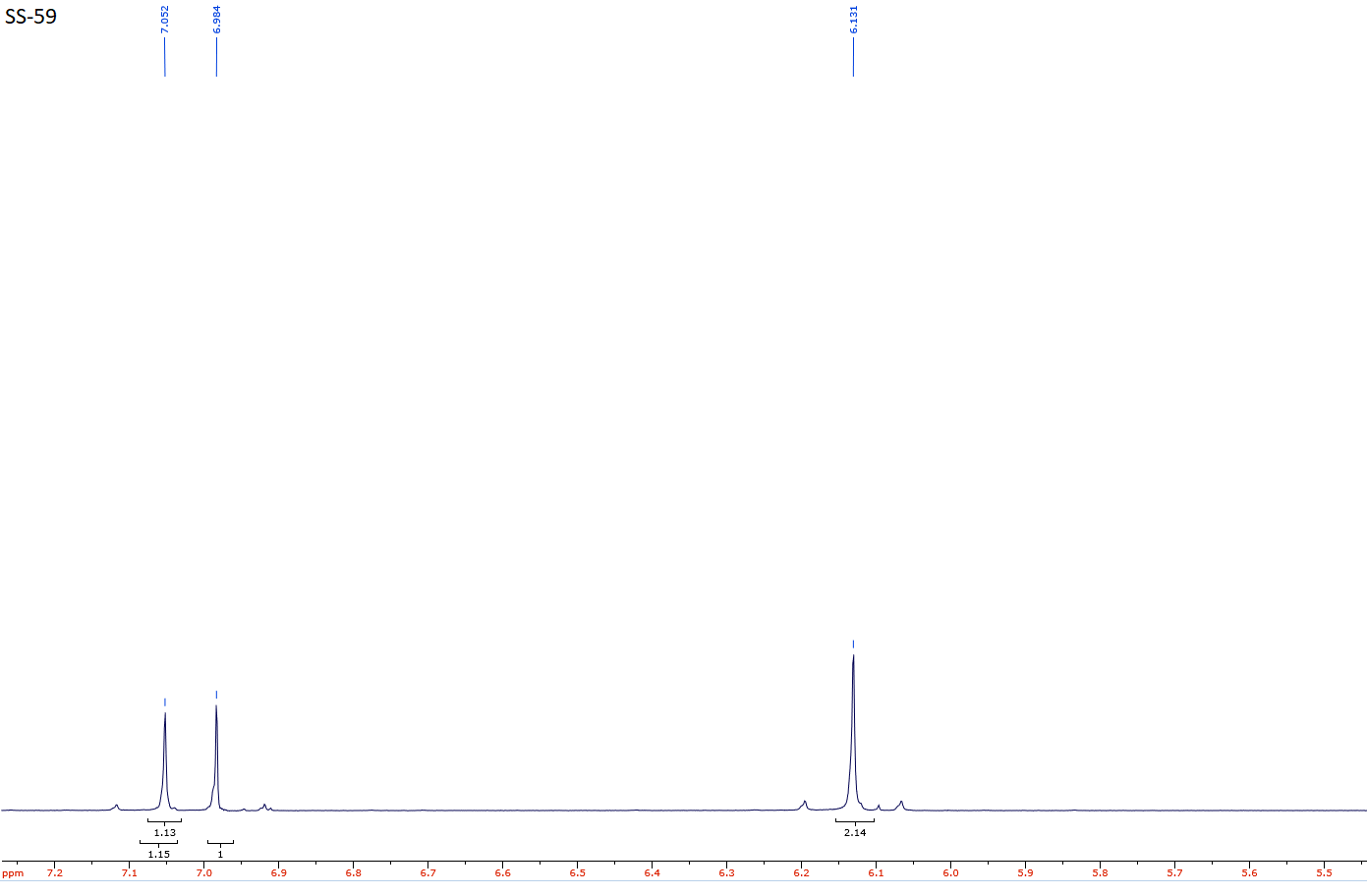


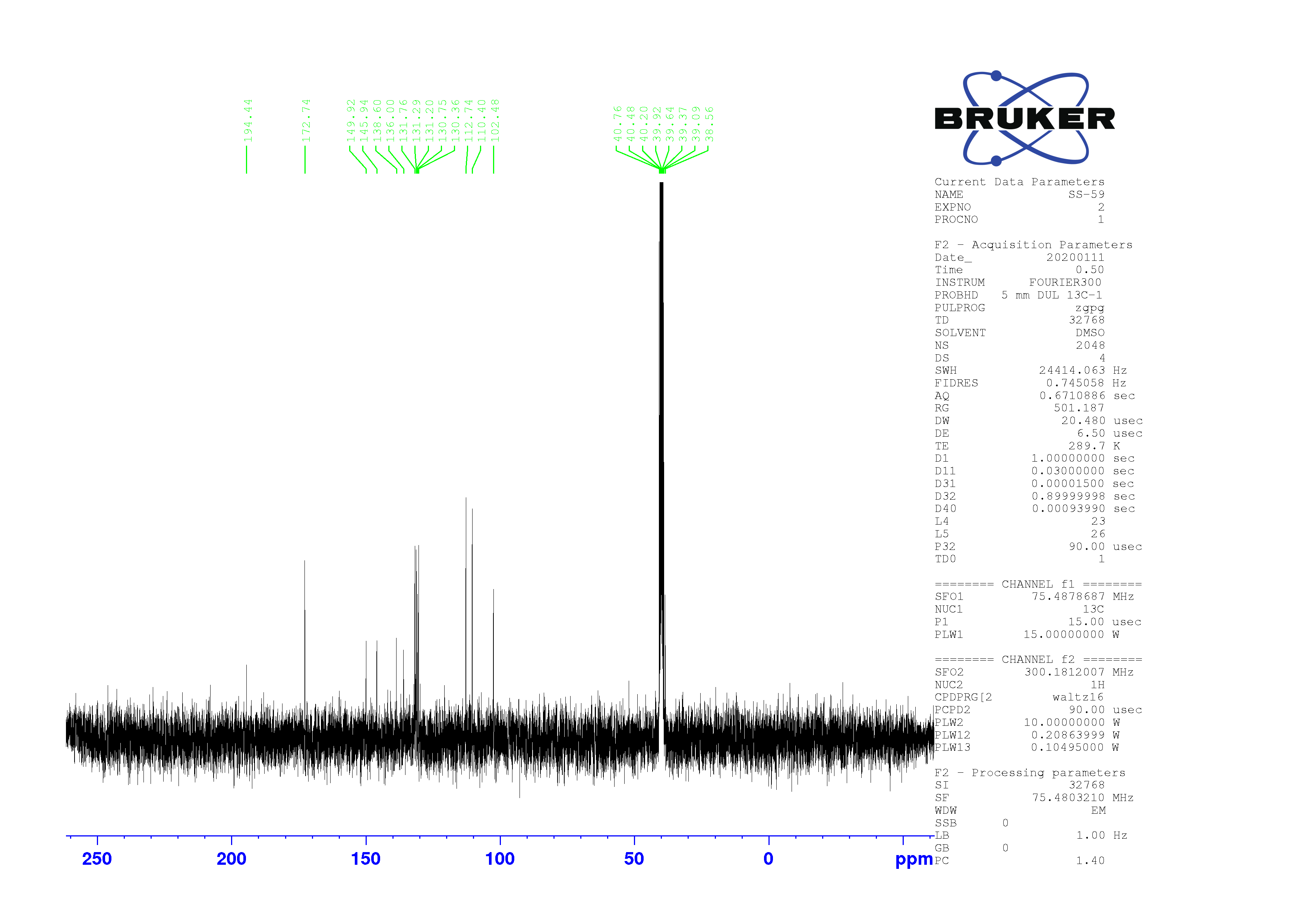


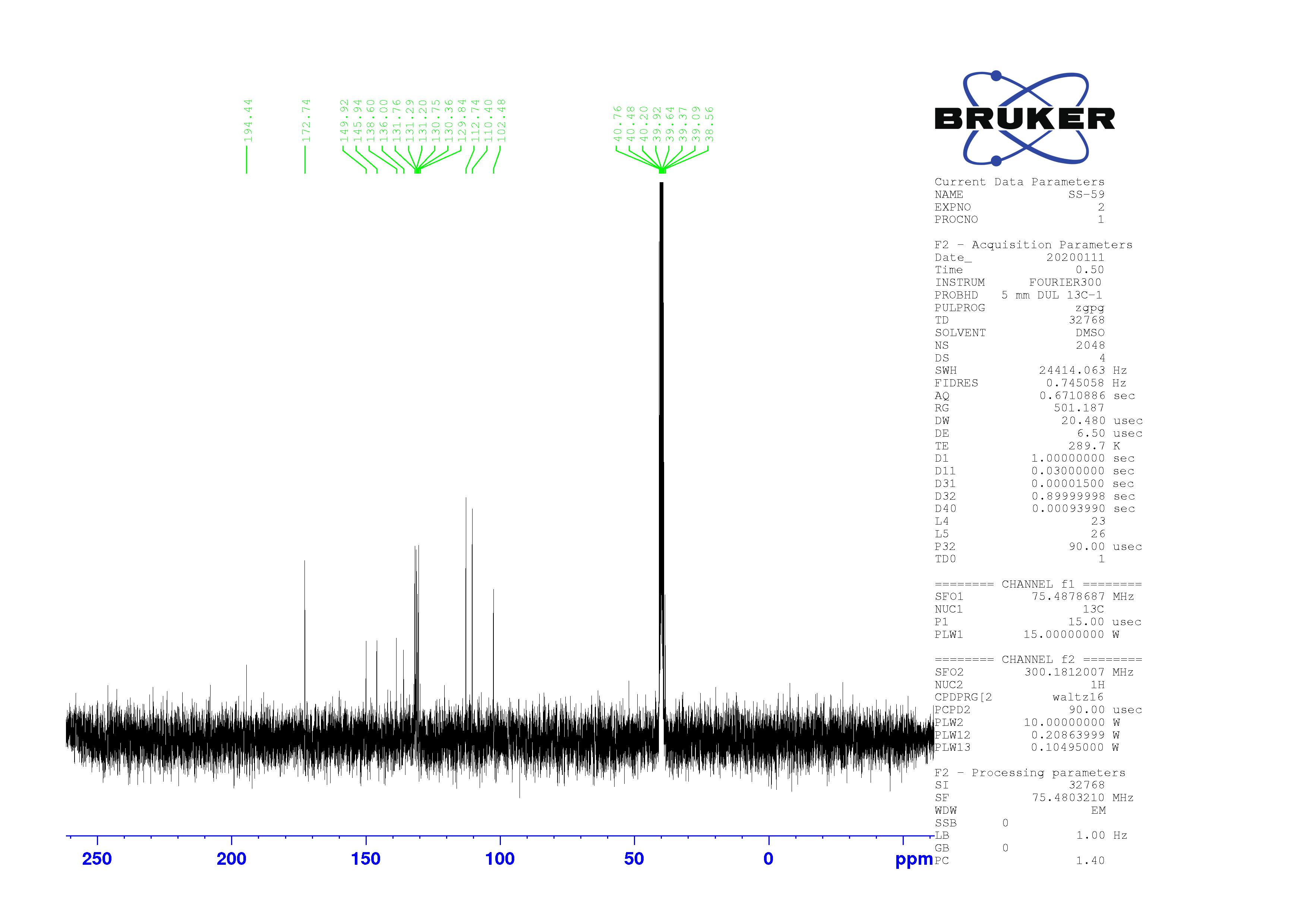


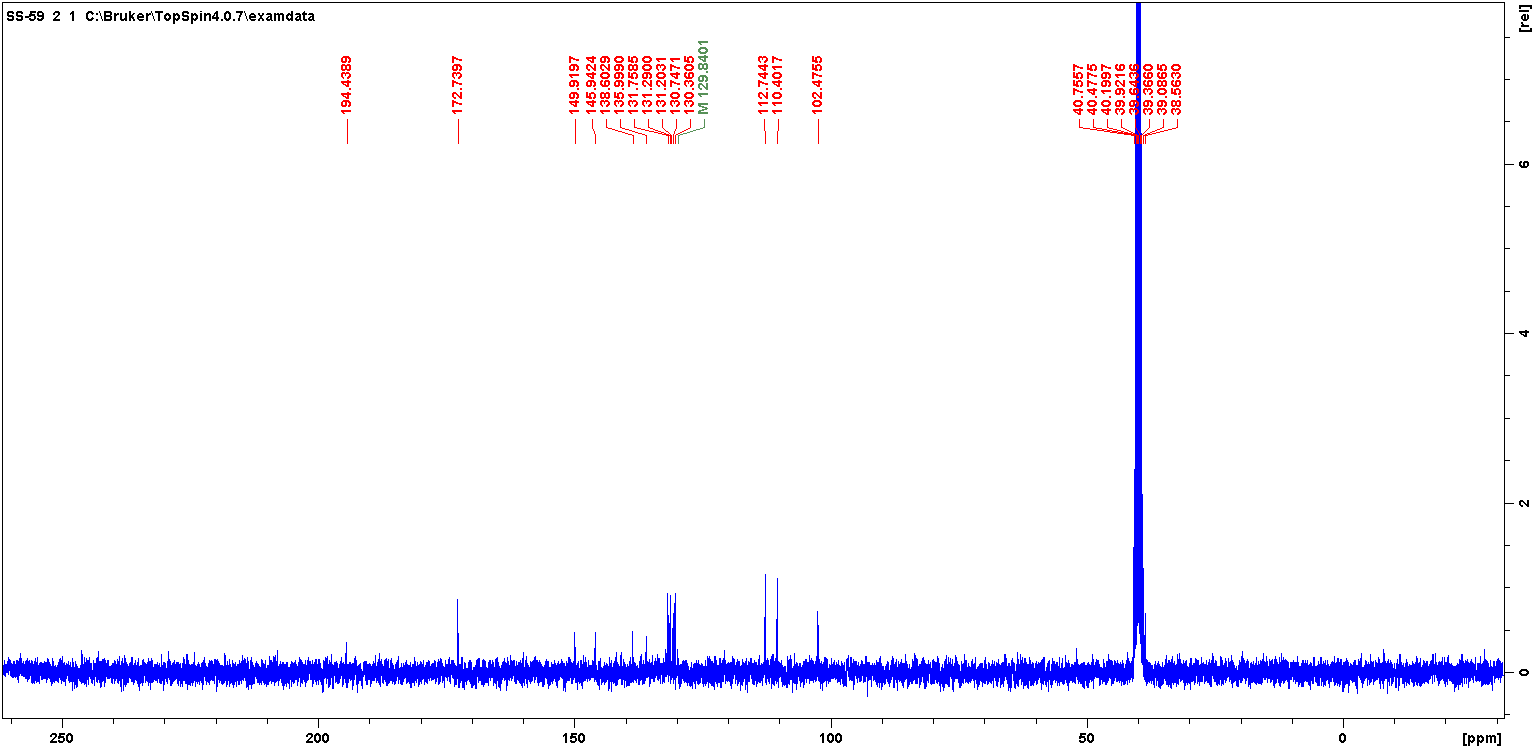


4a


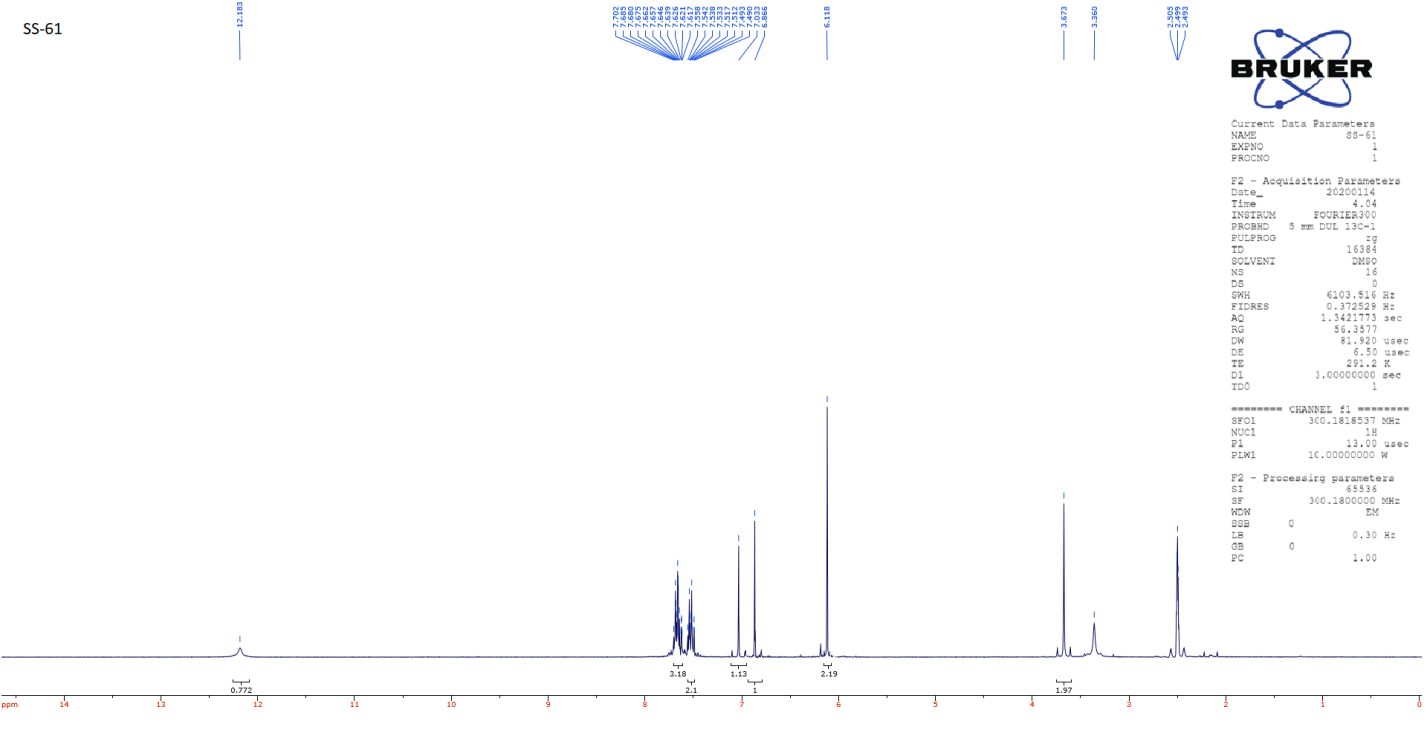


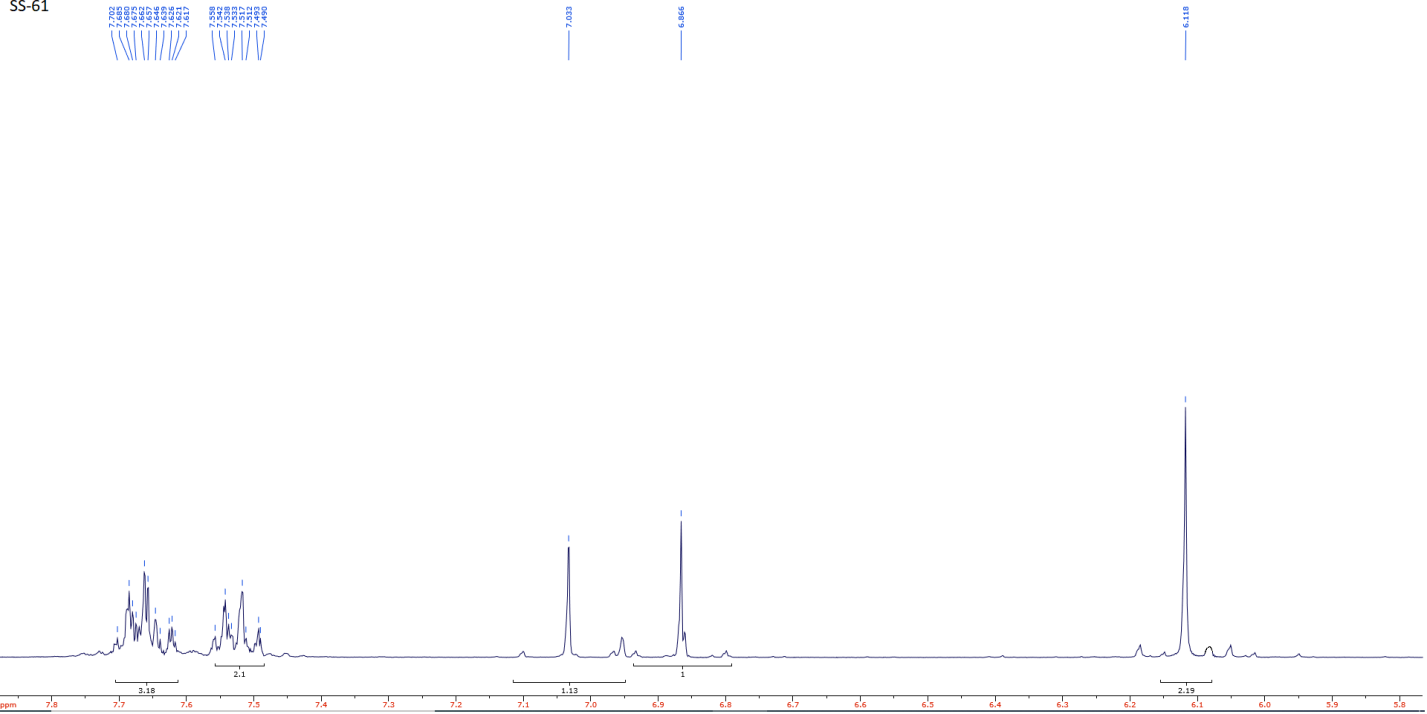


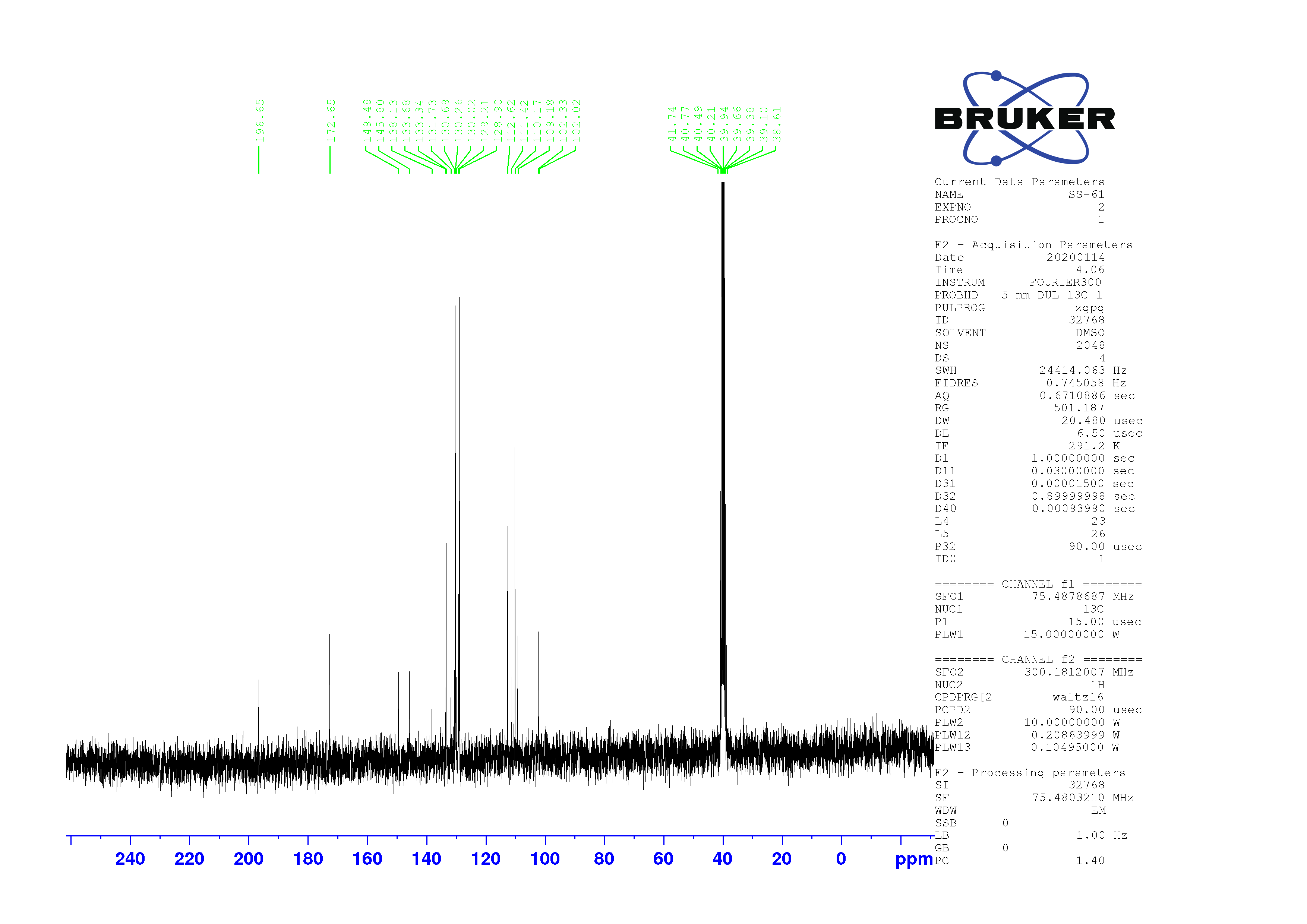

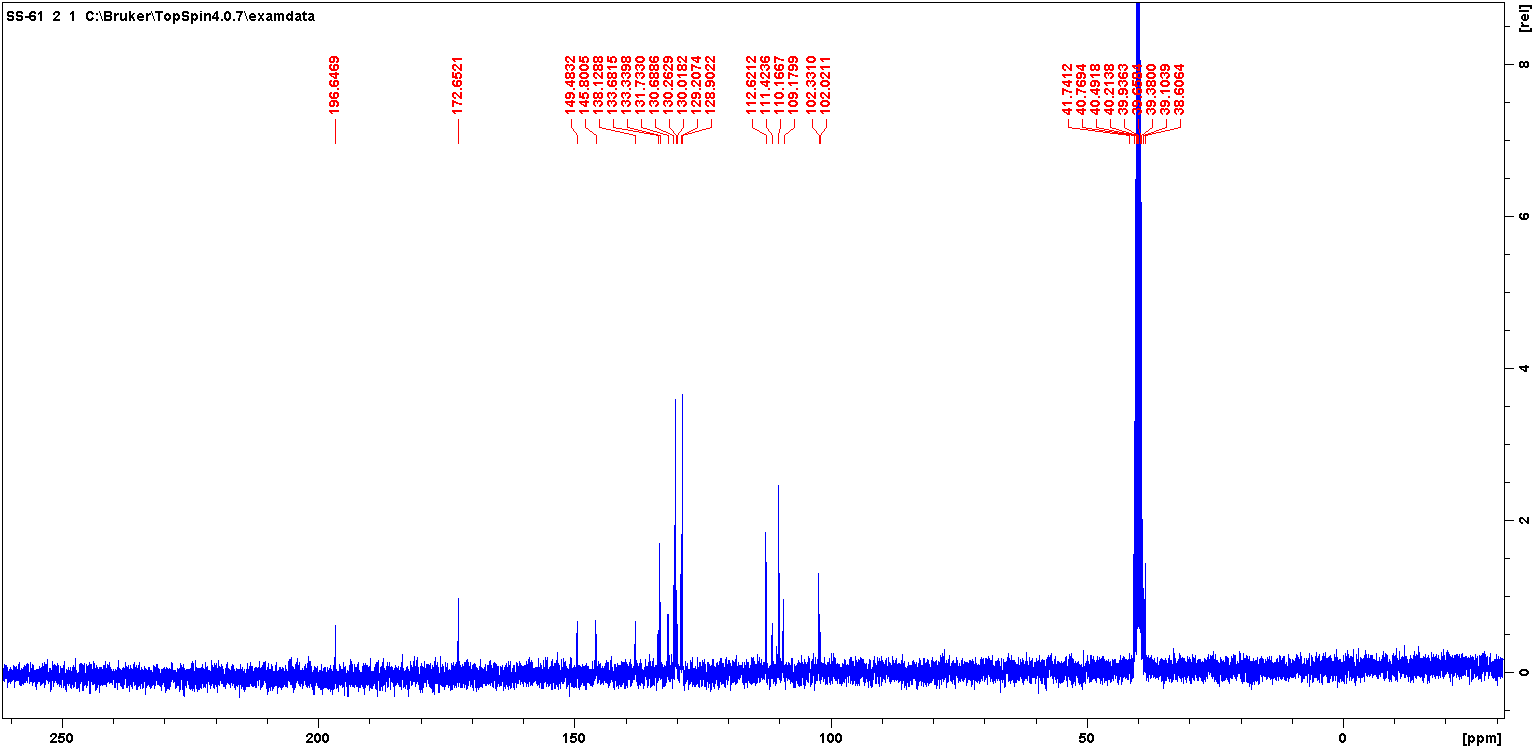


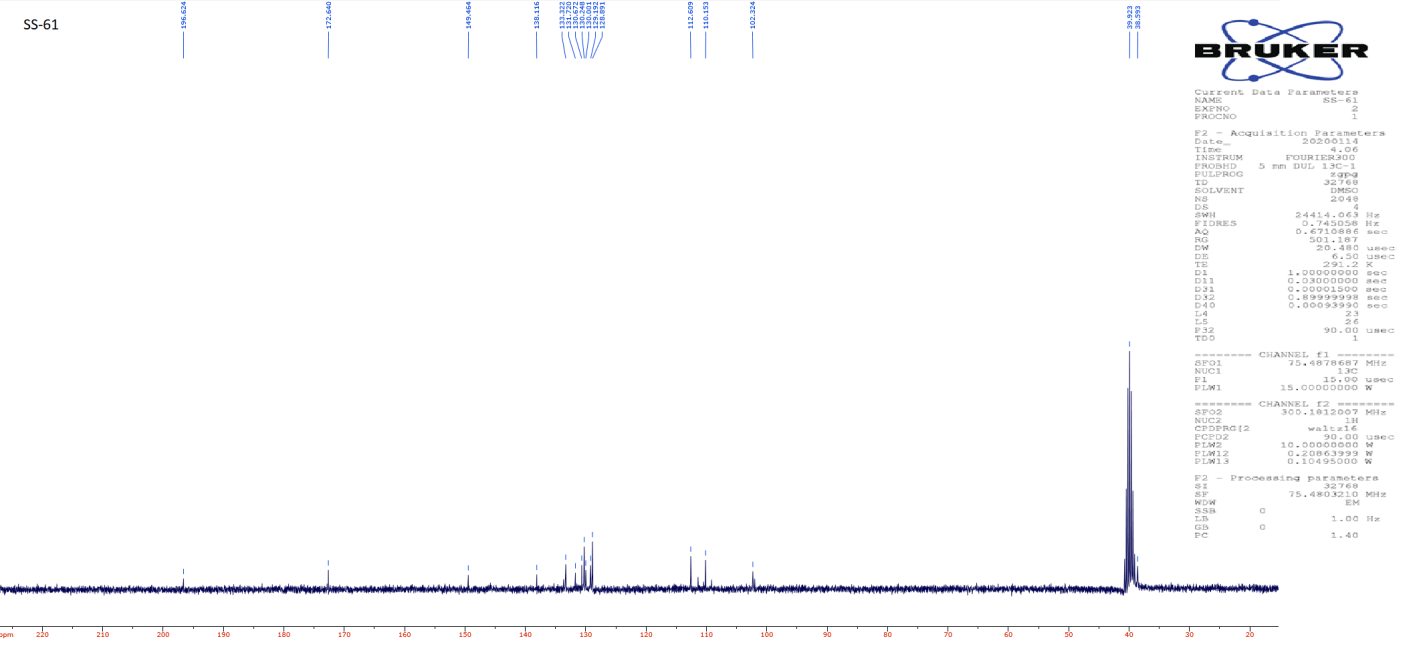

Supplement: Supplementary file 1 — Additional file 1: The data in the addtional file include NMR spectrum files and HRMS file of all newly synthesized compounds described in this article. [file 13065_2020_706_MOESM1_ESM.docx]
